# Genomic characterization reveals distinct mutational landscapes and therapeutic implications between different molecular subtypes of triple-negative breast cancer

**DOI:** 10.1038/s41598-024-62991-3

**Published:** 2024-05-29

**Authors:** Ruo Qi Li, Lei Yan, Ling Zhang, Hai Xia Ma, Hui Wen Wang, Peng Bu, Yan Feng Xi, Jing Lian

**Affiliations:** 1 Department of Pathology, Shanxi Province Cancer Hospital/Shanxi Hospital Affiliated to Cancer Hospital, Chinese Academy of Medical Sciences/Cancer Hospital Affiliated to Shanxi Medical University, Taiyuan, China; 2 General Surgery Department, Shanxi Bethune Hospital, Tongji Shanxi Hospital, Shanxi Academy of Medical Sciences, Third Hospital of Shanxi Medical University, Taiyuan, 030032 China; 3 Shanxi Key Laboratory of Bone and Soft Tissue Injury Repair, Department of Orthopedics, The Second Hospital of Shanxi Medical University, 382 Wuyi Road, Taiyuan, Shanxi China; 4 Department of Pathology, Shanxi Medical University, Taiyuan, Shanxi 030001 China

**Keywords:** Triple-negative breast cancer, Targeted next-generation sequencing, Genomic characterization, Therapeutic implications, Breast cancer, Cancer genomics

## Abstract

Triple-negative breast cancer (TNBC) has high heterogeneity, poor prognosis, and limited treatment success. Recently, an immunohistochemistry-based surrogate classification for the “Fudan University Shanghai Cancer Center (FUSCC) subtyping” has been developed and is considered more suitable for clinical application. Seventy-one paraffin-embedded sections of surgically resected TNBC were classified into four molecular subtypes using the IHC-based surrogate classification. Genomic analysis was performed by targeted next-generation sequencing and the specificity of the subtypes was explored by bioinformatics, including survival analysis, multivariate Cox regression, pathway enrichment, Pyclone analysis, mutational signature analysis and PHIAL analysis. *AKT1* and *BRCA1* mutations were identified as independent prognostic factors in TNBC. TNBC molecular subtypes encompass distinct genomic landscapes that show specific heterogeneities. The luminal androgen receptor (LAR) subtype was associated with mutations in *PIK3CA* and PI3K pathways, which are potentially sensitive to PI3K pathway inhibitors. The basal-like immune-suppressed (BLIS) subtype was characterized by high genomic instability and the specific possession of signature 19 while patients in the immunomodulatory (IM) subtype belonged to the PD-L1 ≥ 1% subgroup with enrichment in Notch signaling, suggesting a possible benefit of immune checkpoint inhibitors and Notch inhibitors. Moreover, mesenchymal-like (MES) tumors displayed enrichment in the receptor tyrosine kinase (RTK)-RAS pathway and potential sensitivity to RTK pathway inhibitors. The findings suggest potential treatment targets and prognostic factors, indicating the possibility of TNBC stratified therapy in the future.

## Introduction

Triple-negative breast cancer (TNBC), accounting for between 15 and 20% of breast carcinomas, is associated with an increased incidence of visceral metastasis as well as a high degree of early recurrence and poor prognosis^1,2^. Although Chemotherapy is the main systemic treatment for TNBC, good responses tend to occur only in the initial stages of the disease^3^. The disease is highly heterogeneous and few molecular targets have been identified, creating significant challenges for the development of specific treatments^4^. Furthermore, responses to the same chemotherapy drugs vary widely among individual patients^5^. Thus, improved therapies and prognostic prediction specifically tailored to TNBC are urgently required.

The classification of TNBC into specific subtypes assists in the development of precisely targeted treatment^6^. Lehmann et al. proposed for the first time to classify TNBC into seven molecular subtypes by clustering gene expression profiles from 21 databases and 587 cases of TNBC: basal-like 1, basal-like 2, immunomodulatory, mesenchymal, mesenchymal stem-like, luminal androgen receptor (LAR); and unstable^7^. Subsequently, Burstein et al. analyzed 198 cases of TNBC using whole genome sequencing method and classified TNBC into four subtypes: LAR, mesenchymal, basal-like immune-suppressed (BLIS), and basal-like immune activating, which was found to better show the relationship between each of the subtypes and prognosis^8^. Lehmann subtyping and Burstein subtyping are both more successful cases of molecular typing of TNBC, depicting the molecular features of TNBC and suggesting possible treatment strategies. However, both have not been verified by clinical trials to confirm the validity of subtyping. In 2019, Shao et al. classified TNBC in China into four subtypes according to mRNA expression of specific genes was proposed; this is the “Fudan University Shanghai Cancer Center (FUSCC) subtyping”^9^. This scheme defines the four subtypes as (1) LAR, (2) immunomodulatory (IM), (3) BLIS, and (4) mesenchymal-like (MES)^9^. Unlike the other two subtypings mentioned above, all the TNBC cases included in the FUSCC subtyping were from East Asian populations, demonstrating for the first time that the molecular characteristics of TNBC have some similarities and differences between different races, and that the FUSCC subtyping better characterizes the genotypic phenotype of TNBC patients in China. Shao et al. are also successively conducting FUTURE clinical trials to investigate the benefits of FUSCC subtyping for personalized treatment of TNBC in the clinic^10^.

To be mentioned, one drawback of transcriptomics is its high expense^11^. The relatively simple and straightforward immunohistochemistry (IHC) staining remains the preferred technique in many laboratories and hospitals^12^. Thus, we developed and validated a surrogate IHC-based classification system using androgen receptor (AR), cluster of differentiation 8 (CD8), and forkhead box C1 (FOXC1) for FUSCC subtyping^13^. Previous studies have shown that there was a great agreement between the IHC-based classification and mRNA-based classification (The overall Cohen’s κ coefficient = 0.678)^14^. Herein, the genomic profiling of TNBC specimens collected after surgical resection were analyzed using targeted next-generation sequencing (NGS) to identify the specific molecular characteristics of the different subtypes and their clinical relevance.

## Results

### Characteristics of enrolled patients

**Figure 1:**
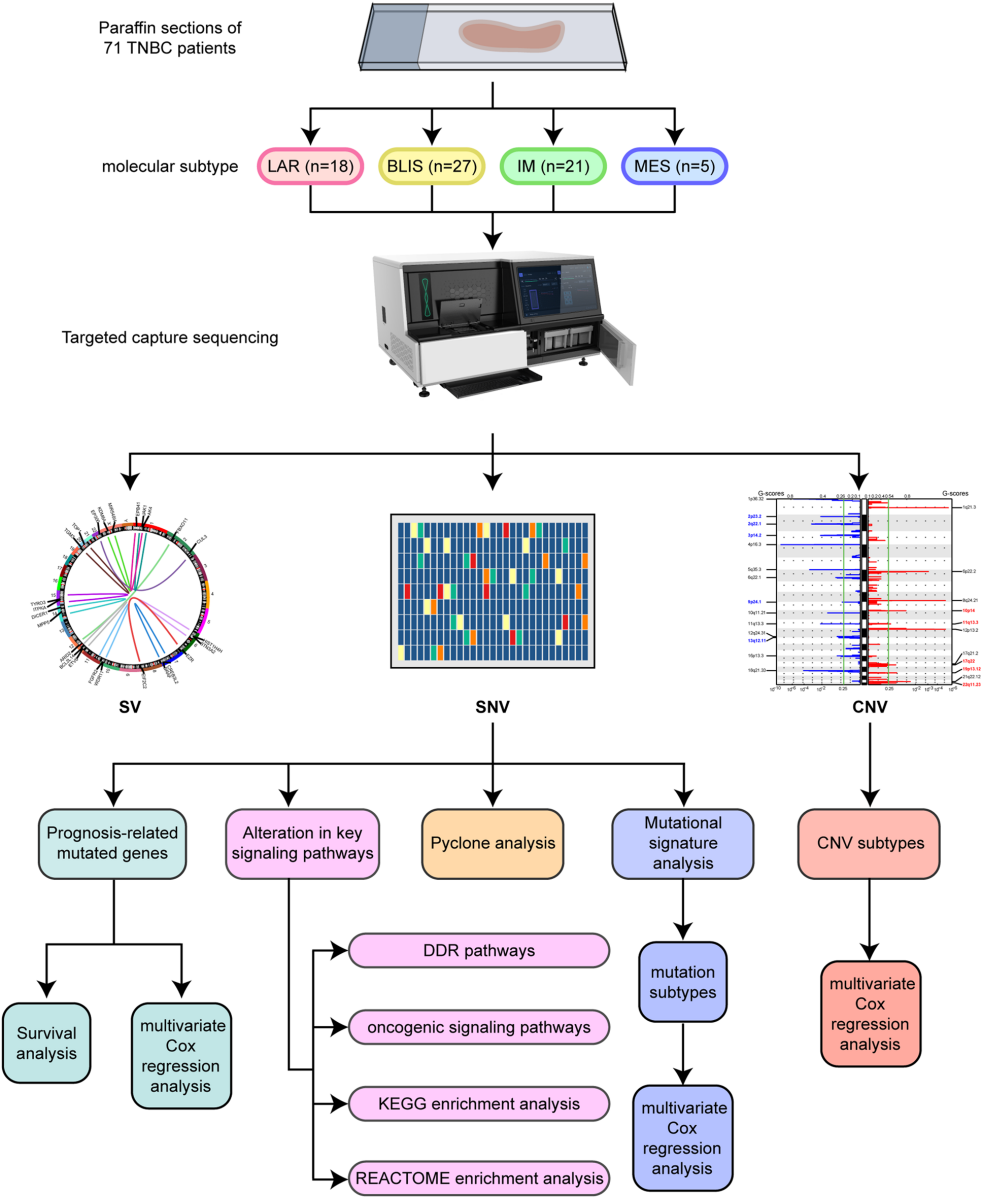
Flow chart of the study. LAR, luminal androgen receptor; BLIS, basal-like immune-suppressed; IM, immunomodulatory; MES, mesenchymal-like; SV, structural variations; SNV, single nucleotide variants; CNV, copy number variations; DDR, DNA damage response.

Overall, 1021 cancer-related genes from tumor samples from 71 patients with TNBC who had undergone surgery in the Department of Breast Surgery, Shanxi Cancer Hospital (SXCH), between January 1, 2017, and December 31, 2019, were analyzed by targeted NGS (Table S2). The study flow chart is illustrated in Fig. 1. None of these patients had received neoadjuvant treatment prior to surgery. All patients underwent surgical treatment according to standard treatment protocols, and received anthracycline-based chemotherapy for four to six cycles. The median age at diagnosis was 51.6 (range, 40–86) years. The number of patients in each subtype were as follows: LAR (n = 18), BLIS (n = 27), IM (n = 21), and MES (n = 5). Table S3 shows details of the clinicopathological features of the patients. The median TMB, the number of nonsynonymous SNVs and indels per megabase, was 6.96 mutations (Mut)/Mb (Table S3). The median DFS of the cohort was 36 months (range, 12.0–57.0 months). No significant associations were observed between age or subtype with DFS. Each tumor sample was observed to contain a minimum of one non-silent mutation or structural variant (Table S4).

### Overall characterization of the SXCH cohort

**Figure 2:**
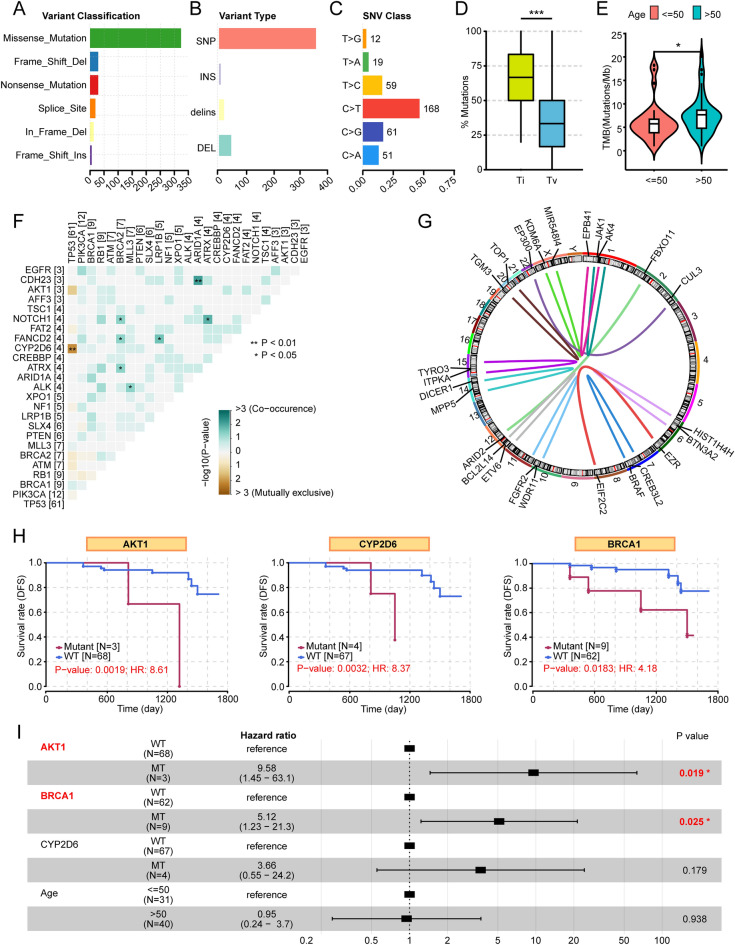
Overall characterization of the SXCH cohort. (**A**) Variant classifications in the SXCH cohort. (**B**) Variant types in the SXCH cohort. (**C**) SNV classes in the SXCH cohort. (**D**) Boxplot showing frequencies of transitions and transversions in the SXCH cohort. (**E**) TMB values in relation to age in the SXCH cohort. (**F**) Pairwise mutual exclusivity and co-occurrence in the SXCH cohort. (**G**) Gene fusions in patients with TNBC. Gene fusions are seen between genes corresponding to the ends of each curve. (**H**) Kaplan–Meier analysis of DFS in patients with and without mutations in AKT1, CYP2D6, and BRCA1. (**I**) Forest plot showing results of the multivariate Cox proportional-hazard regression of AKT1 and BRCA1 mutations and clinical features. Ti, transition; Tv, transversion; DFS, disease free survival; TMB, tumor mutation burden. “*” represents that p-value < 0.05; “**” represents that p-value < 0.01, and “***” represents that p-value < 0.001.

Most of the SNVs observed in the SXCH cohort were missense, followed by frameshift deletions and nonsense mutations (Fig. 2A). The majority of the variants were SNPs (Fig. 2B). Furthermore, almost half of the SNVs were found to be C > T, consistent with the trend seen in the TCGA TNBC cohort (Fig. 2C). SNPs can be classified as transitions and transversions. In our cohort, transitions occur more frequently than transversions (Fig. 2D). Of note is that patients aged over 50 had a higher TMB (Fig. 2E). Analysis of pairwise mutual exclusivity and co-occurrence indicated that *CDH23*/*ARID1A*, *NOTCH1*/*BRCA2*, *NOTCH1*/*ATRX*, *FANCD2*/*BRCA2*, *FANCD2*/*LRP1B*, *ATRX*/*BRCA2*, *A*LK/PTEN showed significant co-occurrence (p < 0.05), and TP53 showed exclusivity with CYP2D6 (p < 0.01), indicating a multiplicity of interactions among driver genes in TNBC (Fig. 2F). With regard to SV, thirteen gene fusions were observed over the 71 samples, of which three were trans-chromosomal fusions (Table S5). The most frequent fusions (2/13) were seen in JAK1. The JAK1-EPB41 fusion and the JAK1-AK4 (intergenic) fusion were identified, indicating driver gene JAK1 may have a significant impact on the development of TNBC (Fig. 2G). To assess somatic CNVs, 18 arm-level and 57 focal CNVs were found in the 71 TNBC samples (Table S6).

Mutations in the dominant cancer-associated genes that were significantly linked with DFS were analyzed. The KM analysis showed that patients with mutations in *AKT1* had markedly shorter DFS (p = 0.0019), and similar results were found for patients with *CYP2D6* (p = 0.003) and *BRCA1* (p = 0.018) mutations (Fig. 2H). Further multivariate Cox regression showed that the presence of mutations in *AKT1* and *BRCA1* were independent predictors of prognosis (Fig. 2I). The mutations in both genes in the SXCH cohort were annotated using lollipop plots (Fig. S1).

### Genomic profiles of molecular subtypes of TNBC

**Figure 3:**
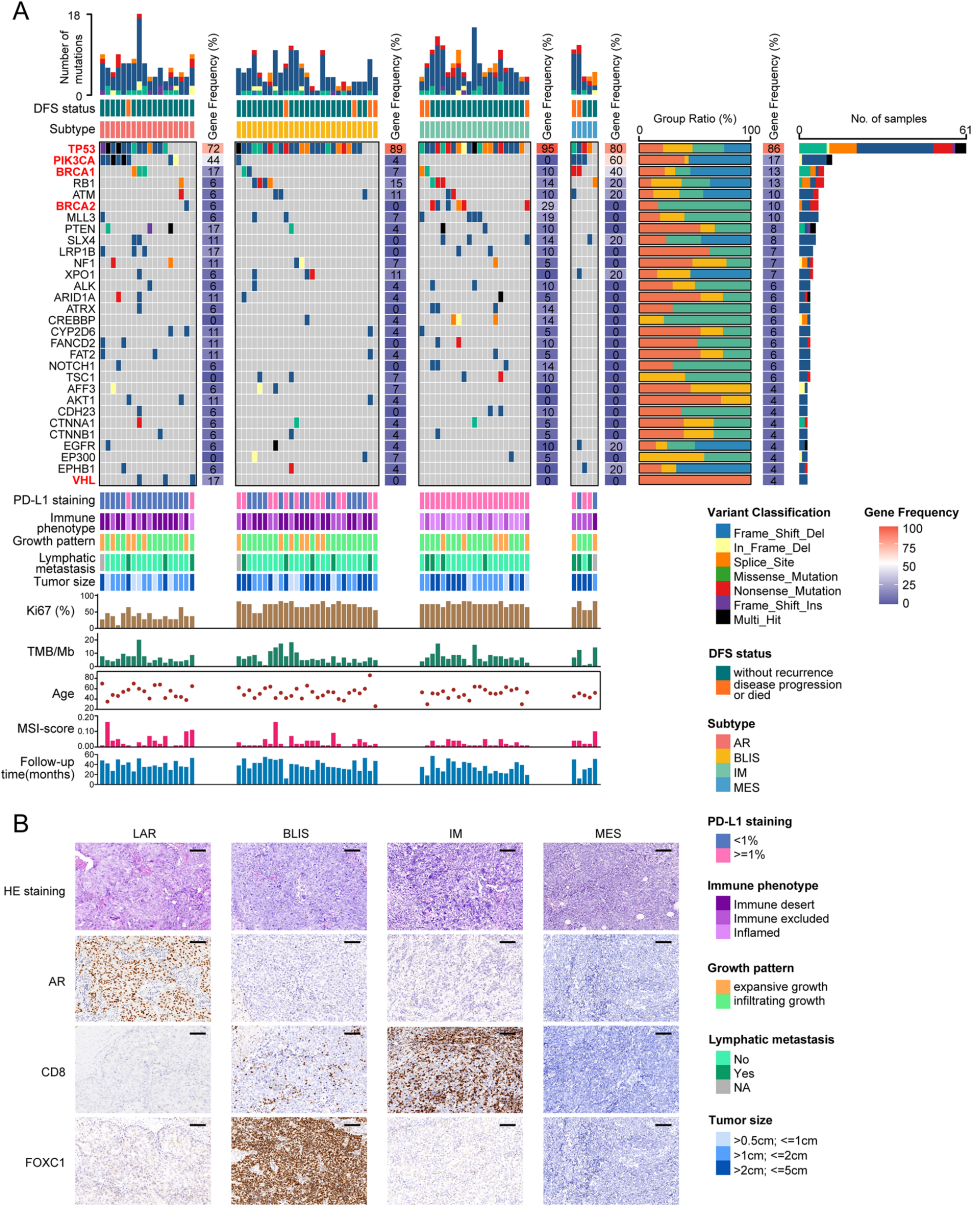
Comparison of the mutational landscapes of the four molecular subtypes. (**A**) Heatmap showing the 30 genes with the highest frequencies of mutation. Columns represent individual samples. The 71 samples were classified into four subtypes and are shown separately. Numbers alongside the heatmap indicate mutation frequencies. Group proportions are shown in the percentage stack histogram on the right. The bar on the far right indicates the classification of genetic variations. (**B**) H&E and IHC staining of the four molecular subtypes. Scale bar = 100 μm. LAR, luminal androgen receptor; BLIS, basal-like immune-suppressed; IM, immunomodulatory; MES, mesenchymal-like; DFS, disease free survival; TMB, tumor mutation burden; MSI, microsatellite instability.

The mutational landscape varied substantially across the molecular subtypes (Fig. 3A). Figure 3B illustrates H&E staining and IHC for the four subtypes (Fig. 3B). Overall, mutations in TP53 were seen most frequently in all four subtypes while mutations in *PIK3CA* occurred most frequently in LAR patients compared with the other subtypes. The *BRCA2* gene showed more frequent mutation (29%) in IM (p < 0.001), while mutations in *BRCA1* (40%) were more often seen in MES than in the other subtypes (p < 0.001). *VHL* mutations showed an overall low prevalence (4%) in the entire cohort, with all three *VHL* mutations observed in LAR-type patients (Fig. 3A). Notably, patients with the IM subtype all fell into the PD-L1 ≥ 1% subgroup. We then investigated the variations in mutations in the top 15 most frequently mutated genes over the different clinical subtypes. This showed that mutations in *BRCA2* and *ATRX* mutations, which occur predominantly in IM patients, were also significantly enriched in the inflamed and PD-L1 ≥ 1% groups (Fig. S2A). All the *ALK* mutations were observed in the group with infiltrating growth (Fig. S2A). The TMB, however, did not differ significantly among the four molecular subtypes (Fig. S2B). Figure S2C shows the distribution of the molecular subtypes in the TMB-H and TMB-L groups.

### Somatic CNVs of different molecular subtypes

**Figure 4:**
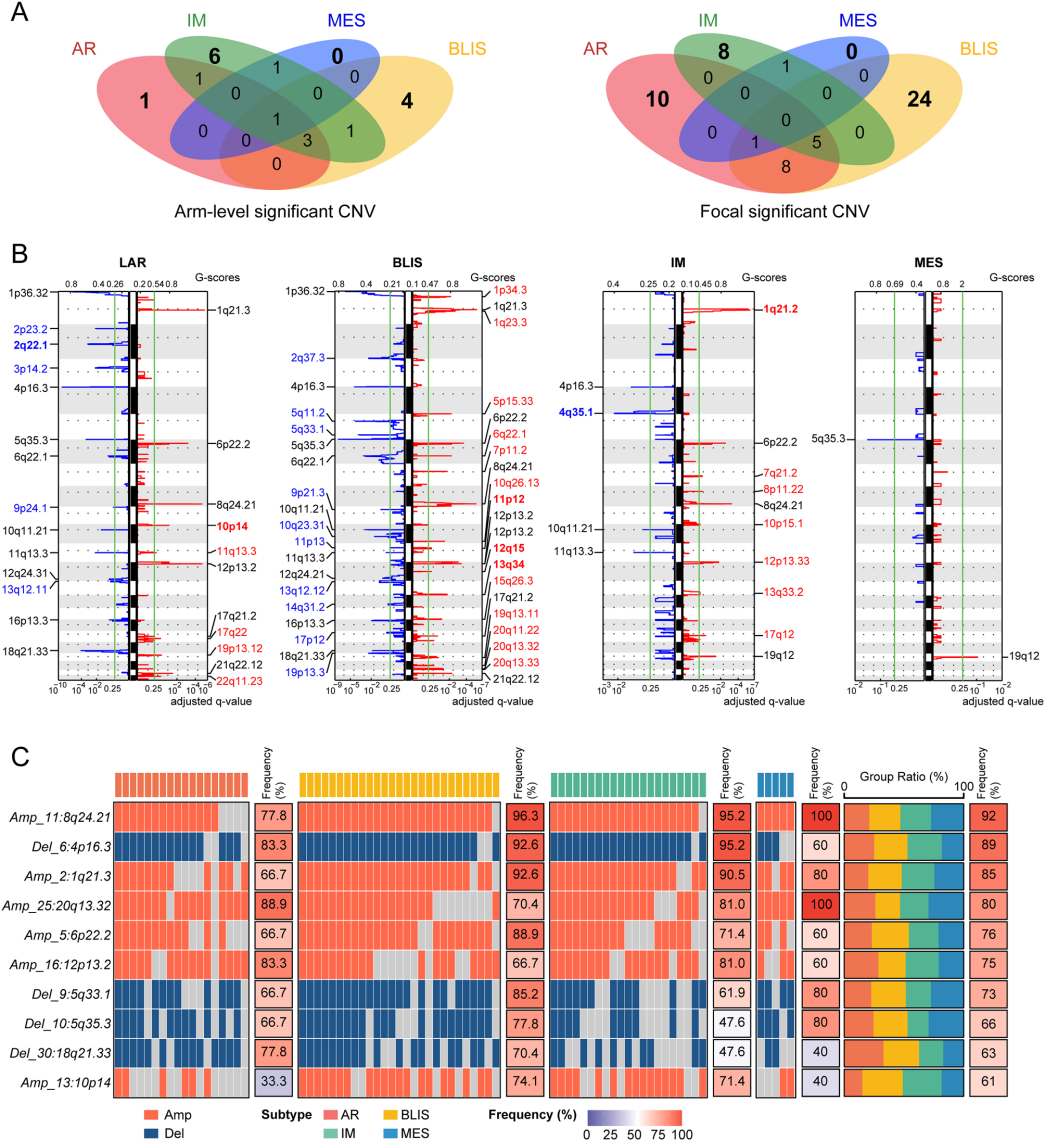
Significantly altered somatic CNVs in TNBC subtypes. (**A**) Venn diagram showing significant arm-level and focal CNVs in TNBC subtypes. (**B**) Significant somatic CNVs in the LAR, BLIS, IM, and MES subtypes. The green line represents the threshold Q-value = 0.25. Significantly amplified focal peaks are shown in red and significantly deleted focal peaks are shown in blue. (**C**) Heatmap showing regions with the greatest CNV frequency. Columns represent individual samples. The 71 samples in the cohort are shown according to their molecular subtype. Numbers alongside the heatmap indicate mutation frequencies. Subtype proportions are shown on the right of the percentage stack histogram. CNV, copy number alterations; Amp, amplification; Del, deletion; LAR, luminal androgen receptor; BLIS, basal-like immune-suppressed; IM, immunomodulatory; MES, mesenchymal-like.

The CNVs varied significantly across the four molecular subtypes (Fig. 4A). Notably, the amplified region with the highest G-score was observed on chromosome 10p14, while the deleted region with the highest G-score was situated on chromosome 2q22.1 in the LAR subtype. The BLIS subtype was found to have the highest number of amplification and deletion regions, suggesting a high level of genetic instability. Moreover, in the IM subtype, the specific amplified region with the highest G-score was located on chromosome 1q21.2 whereas the only specific deleted region was located on chromosome 4q35.1. No specific amplification or deletion regions were found for the MES subtype in the SXCH cohort, which may be related to the small sample size (Fig. 4B). Figure S3 shows bubble plots of the numbers of genes, sample sizes, and significance levels of changes in different regions for each subtype, while Fig. 4C illustrates the top 10 regions showing the highest CNV numbers and their frequency of occurrence in the different molecular subtypes.

### Key signaling pathways affected by SNVs

**Figure 5:**
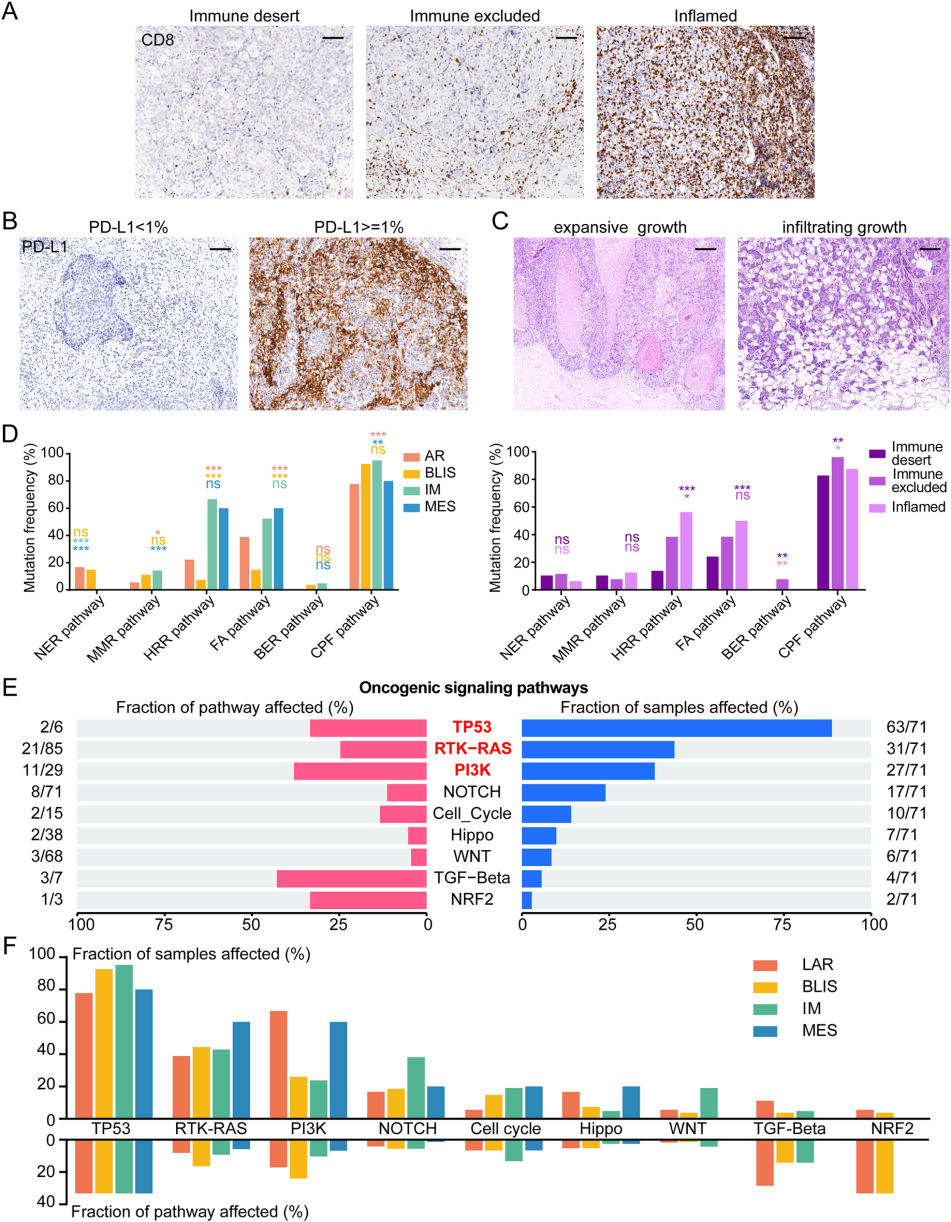
Key signaling pathways affected by somatic mutations in TNBC. (**A**) IHC of distinct immune phenotypes. Scale bar = 100 μm. (**B**) IHC of distinct PD-L1-staining groups. Scale bar = 100 μm. (**C**) H&E staining of distinct growth-pattern groups. Scale bar = 250 μm. (**D**) Mutations in DDR-related pathways in the different subtypes. (**E**) Proportions of affected oncogenic pathways in TNBC samples. (**F**) Proportions of affected oncogenic pathways in TNBC subtypes. NER, nucleotide excision repair; MMR, mismatch repair; HRR, homologous recombination repair; FA, fanconi anemia; BER, base excision repair; CPF, checkpoint factor; LAR, luminal androgen receptor; BLIS, basal-like immune-suppressed; IM, immunomodulatory; MES, mesenchymal-like.

SNVs in DDR-related pathways, which occur relatively frequently in TNBC, have been found to be linked with genomic instability and have been suggested to be associated with better clinical outcomes in patients with TNBC^15^. Eight DDR-related pathways were thus investigated (Table S7). We found that the frequency of DDR pathway mutations varied between different molecular subtypes and clinical groups and the base excision repair (BER) pathway mutations occurred only in the immune-excluded group (Figs. 5A–D, S4A). In addition, ten oncogenic signaling pathways known in TCGA were also analyzed in our study. It was found that most mutations were associated with the TP53 signaling pathway (88.7%), followed by the receptor tyrosine kinase (RTK)-RAS (43.7%) and phosphoinositide 3-kinase (PI3K) (38.0%) pathways (Fig. 5E). It was also observed that most mutations in the PI3K pathways occurred in the LAR subtype (66.7%). Mutations in the Notch pathway were mostly seen in the IM subtype (38.1%) (Fig. 5F). Other clinical subgroups also differed in their oncogenic signaling pathways (Fig. S4B). Enrichment analysis by KEGG and REACTOME indicated the heterogeneity of the TNBC subtypes may be reflected in changes in different key signaling pathways (Fig. S5).

### Clonal and subclonal structural analysis of four molecular subtypes

**Figure 6:**
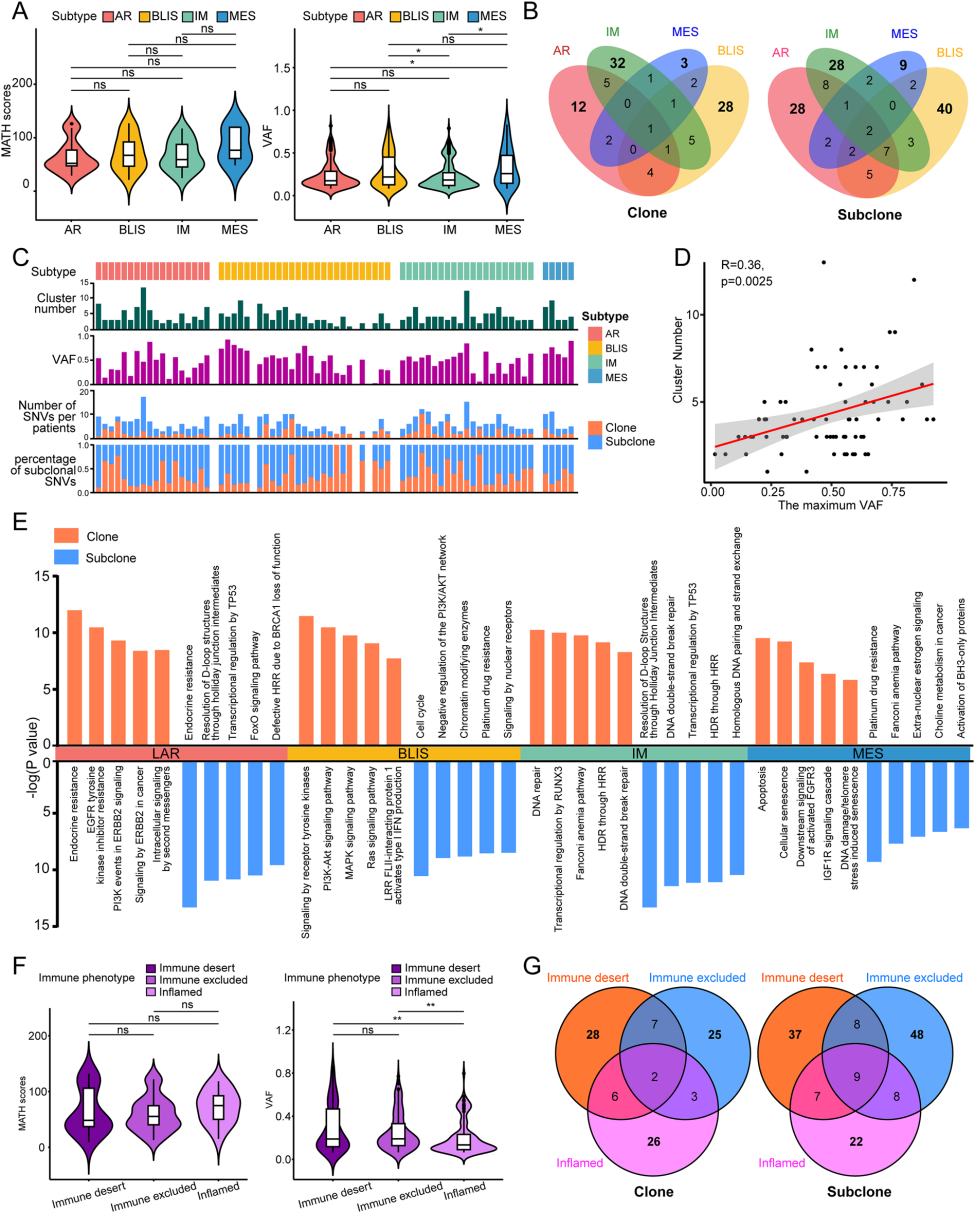
Clonal analysis of TNBC samples. (**A**) Violin plot showing MATH scores and VAF in TNBC subtypes. (**B**) Venn diagrams showing gene numbers in clones and subclones of TNBC subtypes. (**C**) Comprehensive analysis of clonal and subclonal mutation profiles for individual patients. (**D**) Correlations between clone numbers and VAF. (**E**) Enrichment of clonal and subclonal genes in TNBC subtypes. (**F**) Violin plot showing MATH scores and VAF in relation to immune phenotype. (**G**) Venn diagrams showing gene numbers in clones and subclones of immune phenotypes. MATH, mutant-allele tumor heterogeneity; VAF, variant allele frequency; LAR, luminal androgen receptor; BLIS, basal-like immune-suppressed; IM, immunomodulatory; MES, mesenchymal-like; HRR, homologous recombination repair; HDR, homology-directed repair. “ns” represents that p-value > 0.05, “*” represents that p-value < 0.05.

We also compared the MATH scores and VAF in two dimensions, namely, mutational heterogeneity and the frequencies of allelic mutations. We found that although the MATH scores did not differ significantly between the four subtypes, the VAF values did (Fig. 6A). Pyclone analysis was then performed, defining clusters with the highest cell prevalence as clones and others as subclones. Pyclone identified 157 clones (LAR, 36, 22.9%; BLIS, 51, 32.5%; IM, 59, 37.6%; MES, 11, 7.0%) and 273 subclones (LAR, 82, 30.0%; BLIS, 83, 30.4%; IM, 85, 31.1%; MES, 23, 8.4%). The median clone number (CN) was found to be 2 (range, 1–12). Clonal genes with high specificity were found in each type (unique ratio: LAR, 33.3%; BLIS, 54.9%; IM, 54.2%; MES, 27.3%), and a similar phenomenon was observed for subclonal genes (unique ratio: LAR, 34.1%; BLIS, 48.2%; IM, 32.9%; MES, 39.1%) (Fig. 6B). These findings demonstrate specific mutational differences between the four subtypes. The clonal and subclonal mutational profiles for the different patients are shown in Fig. 6C. Additionally, a significant association was observed between CN and the maximum VAF (Fig. 6D).

Pathway enrichment analysis was undertaken to explore the functions of the clonal and subclonal genes (Fig. 6E). This showed that clonal genes in LAR were significantly enriched in ERBB2 signaling, while subclonal genes were associated with transcriptional regulation by TP53. Enrichment of subclonal genes in BLIS suggested that patients with this subtype may be resistant to platinum drugs. Several classical DNA repair pathways were enriched in the IM, such as homology-directed repair through homologous recombination repair, and DNA double-strand-break repair. Clonal genes in MES were associated with both apoptosis and cellular senescence, while subclonal genes were enriched in choline metabolism (Fig. 6E). These differences in functional enrichment between the four subtypes suggest an association between subtypes and the evolution of clonal patterns. The VAF values differed between immune phenotypes but not in other clinical groups, suggesting that VAF may influence the immune phenotype (Figs. 6F–G, S6A). Thus, we investigated the effects of these genetic mutations underlying these differences (Fig. S6B).

### Mutational signature distribution and the prognostic effect of signature 9

**Figure 7:**
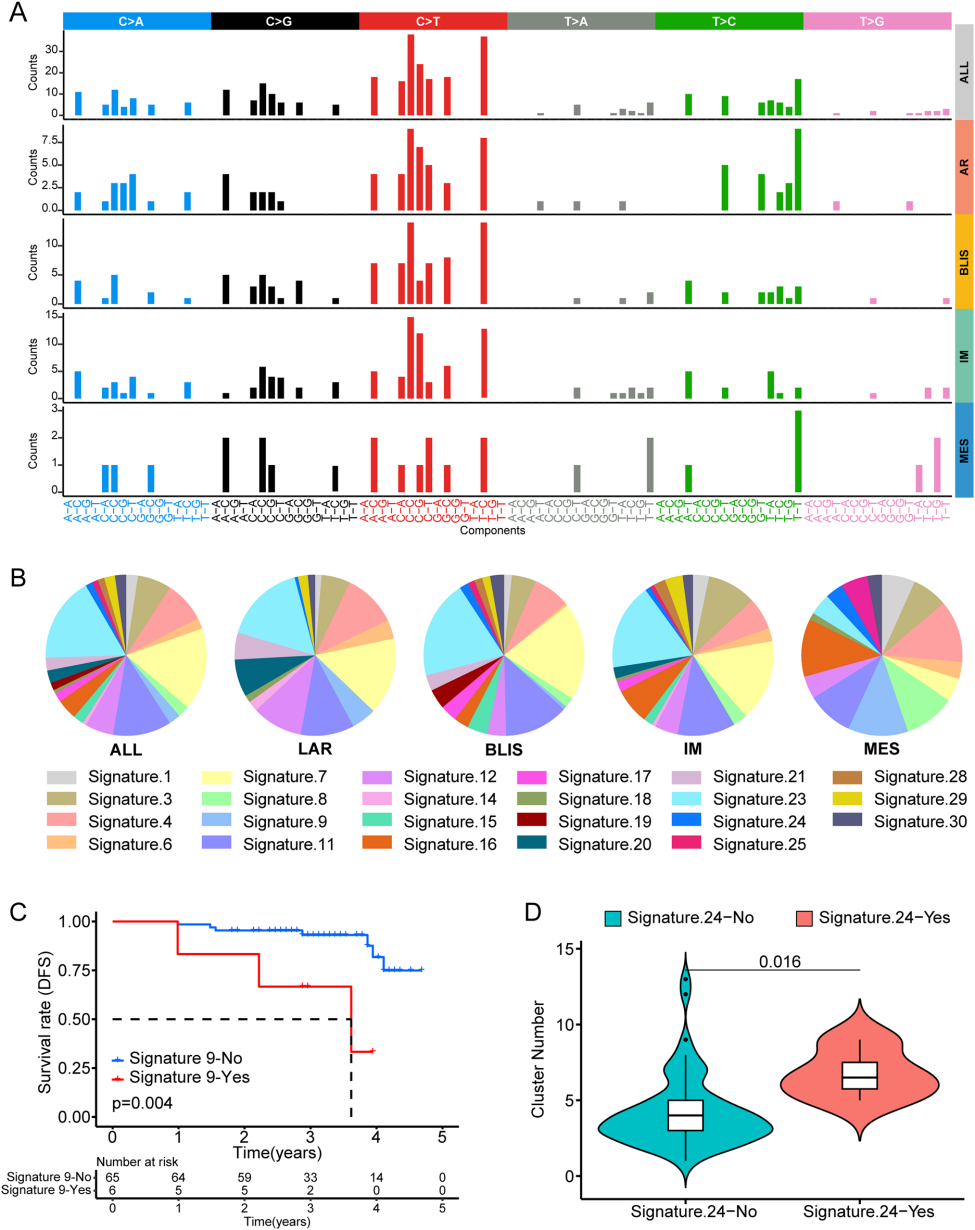
Mutation signatures in TNBC. (**A**) Mutational activities of different molecular subtypes in the SXCH cohort. (**B**) Mutation signatures in LAR, BLIS, IM, and MES subtypes. (**C**) The Kaplan–Meier survival analysis of patients with or without signature 9. (**D**) The relationship between signature 24 and clonality measures (the cluster number). LAR, luminal androgen receptor; BLIS, basal-like immune-suppressed; IM, immunomodulatory; MES, mesenchymal-like; DFS, disease free survival. “*” represents that p-value < 0.05; “**” represents that p-value < 0.01, and “***” represents that p-value < 0.001.

Mutational signatures have been utilized for multiple purposes, including comprehending tumor development, identifying gene alterations associated with mutational processes, and, most importantly, serving as biomarkers for predicting treatment response^16^. The NGS data were analyzed to extract mutational signatures, based on the COSMIC database (Table S8). C > T was found to have a greater frequency (Fig. 7A). Signature 20 was mainly observed in patients with the LAR subtype, while signature 8 was mostly seen in patients with the MES subtype (Fig. 7B), suggesting that LAR-subtype tumors may be associated with mismatch repair (MMR) deficiencies, while MES-subtype tumors may be associated with homologous recombination (HR) deficiencies (Table S8). Remarkably, signature 19 was only observed in patients with the BLIS subtype, although its function is not well understood. In addition, signature 19 was enriched in the immune-excluded, PD-L1-positive ≥ 1%, and the invasive-growth groups (Fig. S7A), suggesting directions for future research into signature 19. The KM curve indicated that patients lacking signature 9 had a more favorable prognosis (Fig. 7C). The differences in mutations between patients with and without signature 9 are shown in Fig. S7B. The top five signatures with the greatest weight (signatures 3, 4, 7, 11, and 23) were investigated (Fig. S7B). Furthermore, the relationship between mutation signature and clonality measures (the cluster number) was explored. The results showed that patients who underwent signature 24 had a greater cluster number compared to patients who lacked signature 24, suggesting that signature 24 is more active in tumors with a large number of subclonal mutations (Fig. 7D). In addition, the "SomaticSignatures" package was also used to explore the mutational signature of TNBC. The results showed that when the number of signatures exceeded 9, the approximation of the residual sum of squares (RSS) and explained variance did not improve significantly with each additional signature, suggesting that TNBC could be categorized into 9 mutational signatures (Fig. S8A–C). Figure S8D illustrates the relative contribution of each signature in different molecular subtypes. Signature S8 was found to be more abundant in AR subtype, while signatures S5 and S9 contributed more in MES subtype. The biological significance behind each signature needs to be further explored in the future.

### TNBC Subtypes based on mutational signature and CNVs

**Figure 8:**
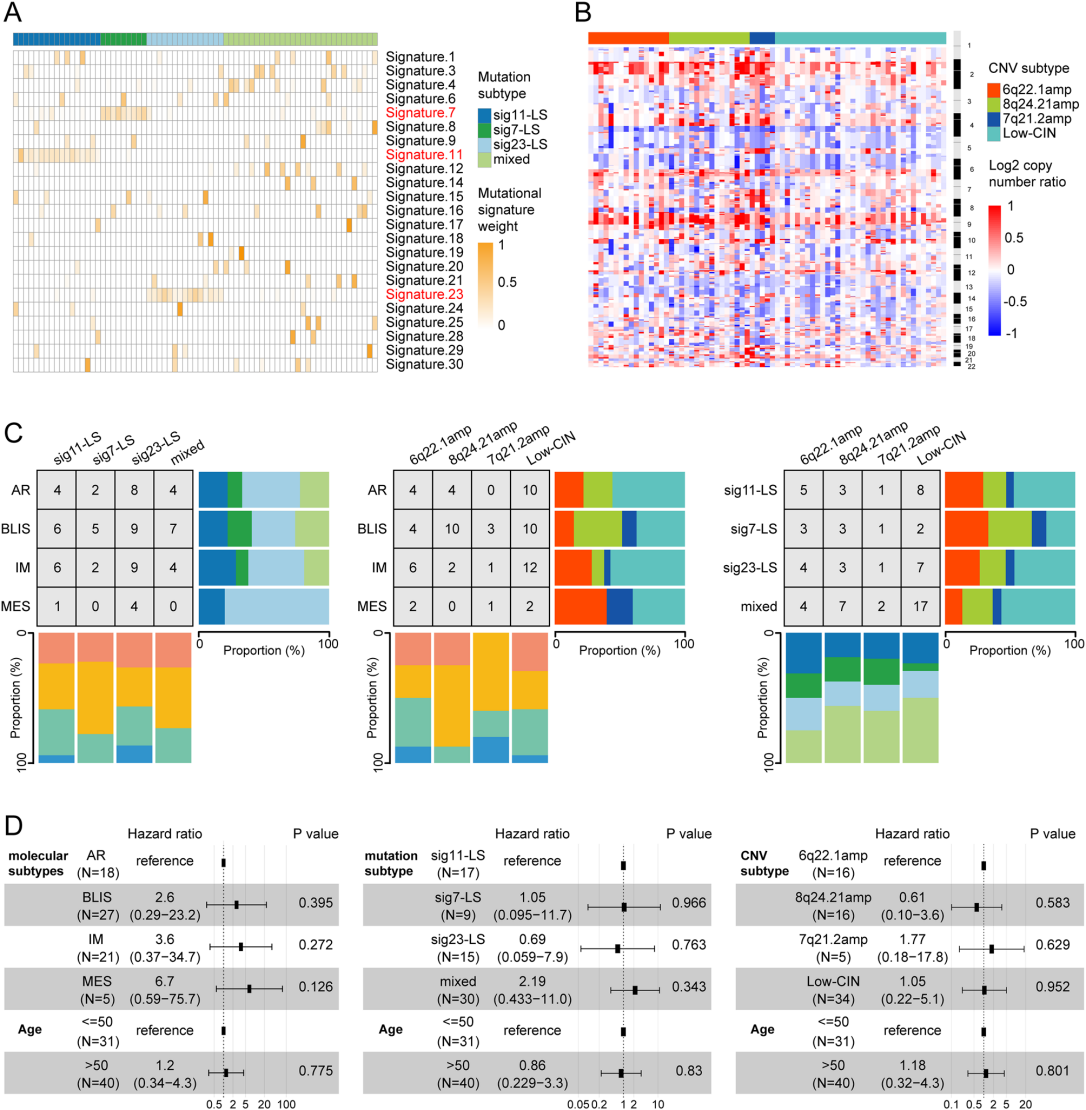
Subtyping of TNBC with mutation signature and CNV data. (**A**) Clustering based on mutation signatures. The heatmap shows the contribution of mutation signatures to the four mutation subtypes. (**B**) Clustering of CNVs shown by GISTIC peaks. Heatmap showing log2 copy-number ratios in the genome. (**C**) Associations between molecular and mutation subtypes (left), molecular and CNV subtypes (middle), and CNV and mutation subtypes (right). (**D**) Associations between molecular (left), mutation (middle), and CNV (right) subtypes with DFS. Cox regression analysis with adjustment for age was used. Hazard ratios are shown with 95% CIs. LAR, luminal androgen receptor; BLIS, basal-like immune-suppressed; IM, immunomodulatory; MES, mesenchymal-like.

Alterations in DNA, such as CNVs and somatic mutations, act as “drivers” promoting tumor growth. They also contain footprints associated with specific biological processes related to tumor growth. We used k-means and consensus clustering, leading to the identification of four clusters based on COSMIC mutational signatures (Fig. 8A). These were mutation subtype 1 (sig11-LS) which was dominated by signature 11, mutation subtype 2 (sig7-LS) which included mainly signature 7, mutation subtype 3 (sig23-LS), including signature 23, and mutation subtype 4, which showed no specifically dominant signature (mixed). Furthermore, four clusters based on CNV peaks were identified (Fig. 8B; Table S9), namely, the CNV subtype 1, with frequent Chr6q22.1 amplification (6q22.1amp), CNV subtype 2, with frequent Chr8q24.21 amplification (8q24.21amp), CNV subtype 3, showing frequent Chr7q21.2 amplification (7q21.2amp), and CNA subtype 4, with low chromosomal instability (low-CIN). To elucidate the relationships between molecular subtypes and mutational signatures, we investigated the specific associations among molecular, CNV, and mutation subtypes. This showed that most of the BLIS-subtype samples (74%) were classified as 8q24.21amp and low-CIN, while the IM and MES subtypes were rarely classified as 8q24.21amp (Fig. 8C). The potential prognostic value of these findings was investigated, revealing that molecular subtypes, CNV, and mutation subtypes were not reliable predictors of DFS (Fig. 8D). The specific associations between molecular subtypes, CNV, and mutation subtypes with clinical groups were examined, and it was found that clinical groups did not predict prognosis reliably (Fig. S9).

### Therapeutic implications of TNBC Subtypes.

**Figure 9:**
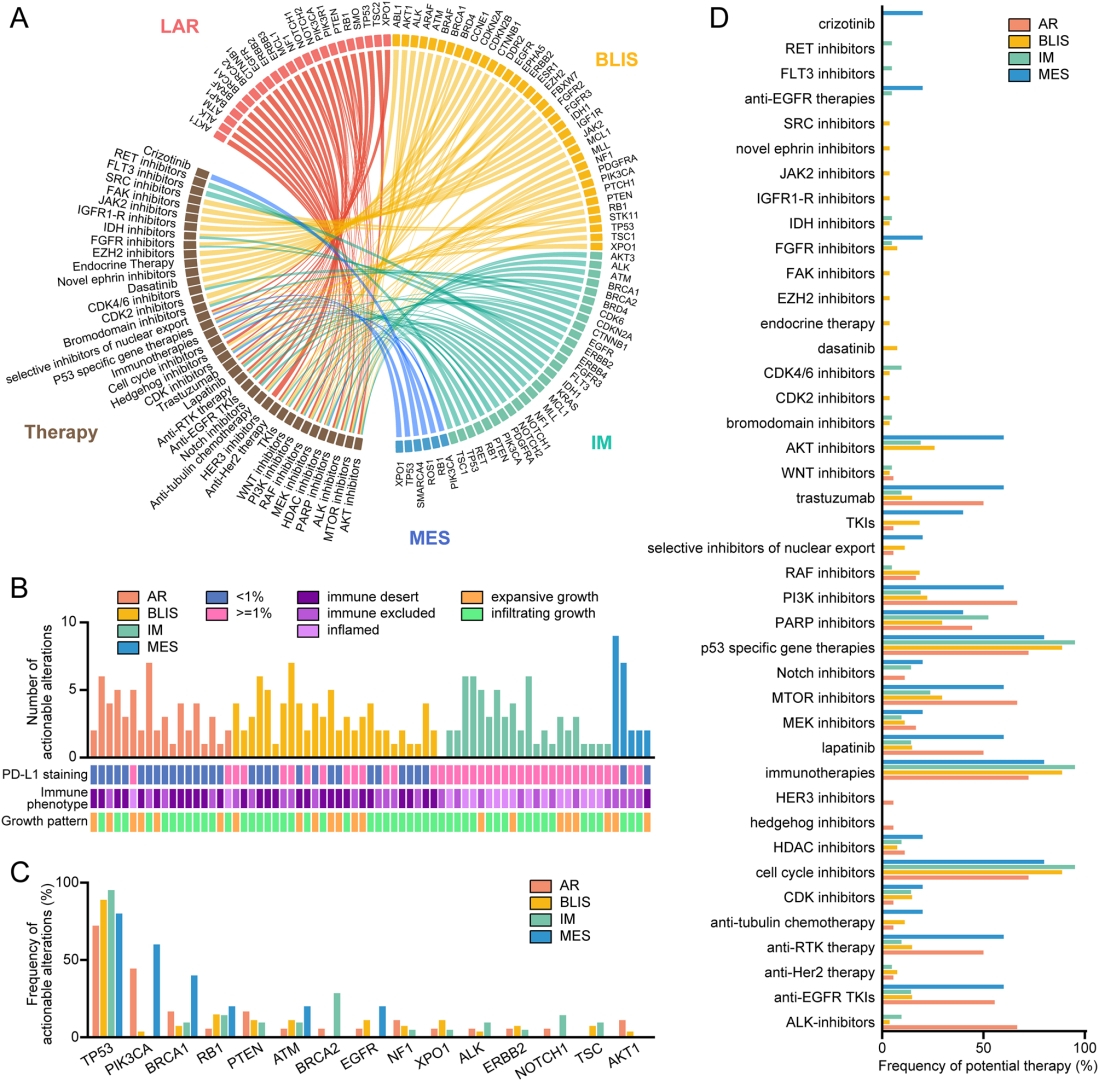
Overview of clinically relevant mutations in 71 TNBC samples. (**A**) Landscape of mutated genes and their potential therapeutic applications in TNBC. Molecular subtypes are indicated by color. (**B**) Numbers of clinically relevant mutations in TNBC molecular subtypes. (**C**) Top 15 genes with greatest numbers of clinically relevant mutations. (**D**) Proportions of patients potentially benefiting or insensitive to therapies specific for subtype-specific TNBC therapy. LAR, luminal androgen receptor; BLIS, basal-like immune-suppressed; IM, immunomodulatory; MES, mesenchymal-like.

In total, 70 (98.5%) patients were found to have at least a single clinically relevant genetic alteration. Alterations that could be targeted, together with possible therapies, are shown in Fig. 9A and Supplementary Table S10. The number of potentially actionable changes varied among patients (median, 3 alterations/patient; range, 0–9 alterations/patient) (Fig. 9B). The molecular subtypes were found to differ in therapeutic implications. Most potentially actionable changes were observed in *TP53* (61/71, 85.9%), *PIK3CA* (12/71, 16.9%), *BRCA1* (9/71, 12.7%), and *RB1* (9/71, 12.7%) (Fig. 9C). *TP53* mutations are considered to have therapeutic implications for cell cycle inhibitors, immunotherapy, and p53-specific gene therapy. In addition, *PIK3CA* mutations are considered to have pharmacological implications for PI3K/rapamycin kinase (mTOR) pathway inhibitors, while *BRCA1* mutations could be targeted by poly (ADP-ribose) polymerase (PARP) inhibitors and *RB1* mutations by cyclin-dependent-kinase (CDK) inhibitors (Table S10). Figure 9D shows the proportions of patients with different molecular subtypes who may benefit from or resist to specific therapies (Fig. 9D). In addition, we observed the potential possibilities of therapeutic agents for different clinical subgroups (Fig. S10).

## Discussion

Treating primary TNBC as a single disease has been found to result in poor therapeutic response^3^. The proposal of molecular subtypes and stratified treatment is an inevitable trend for future research in TNBC^17^. Distinct from Lehmann subtyping and Burstein subtyping, which mainly include European and American populations, the FUSCC subtyping are more suitable for describing the biological characteristics of TNBC patients in East Asia^7,8,17^. The emergence of FUSCC subtyping provides direction for the treatment of TNBC in the clinic. With the need for more portable classifiers in the clinic, an IHC-based surrogate method to FUSCC subtyping have emerged. Previous studies have shown a large agreement between IHC-based and mRNA-based classification (overall Cohen's κ coefficient = 0.678)^14^. Of these, both classification methods showed the highest agreement in categorizing TNBC into LAR subtypes (Cohen's κ coefficient [κ] = 0.821; 95% CI, 0.733–0.908)^14^. In addition, the percentage of samples classified as the same subtype by both classification methods was 76.7%, suggesting that most of the molecular features and therapeutic significance of the mRNA subtypes are retained in the corresponding IHC subtypes. The application of IHC detection of AR has been extensively studied. Clinically, the AR signaling pathway has the potential to be targeted in these LAR tumors^18^. CD8 is a widely studied immune marker, and its expression correlates with patient prognosis and tumor response to immunotherapy^19^. High expression of FOXC1 correlates with invasive tumor biological behaviors and poor prognosis^20,21^. Therefore, it is sufficient to consider clinical interventions based on the classification of IHC.

In contrast to whole genome sequencing (WGS) and whole exome sequencing (WES), targeted NGS focuses on the region of interest, achieving ultra-high sensitivity and accuracy with less data volume, while removing the interference of redundant data and enabling rapid screening of variant loci^22^. With low sequencing cost and deep sequencing depth, targeted NGS has tremendous potential in clinical applications. In this study, IHC was used to classify tumor samples from 71 Chinese TNBC patients into four subtypes and then targeted DNA-NGS was performed on all samples, to determine the relationships between the tumor genetics and the clinical features. This study is the first to explore the association between targeted NGS results, survival outcomes, and the evolution of the TNBC subtypes.

The overall genomic characteristics of TNBC were described in our study. The most prevalent somatic mutations were found to be in *TP53* and *PIK3CA*, with mutations present in 86% and 17% of cases, respectively, followed by *BRCA1* (13%), *RB1* (13%), and *ATM* (10%), It is worth mentioning that the observed frequencies of *TP53* and *PIK3CA* mutations are consistent with previously reported results^17^. Moreover, in the present study, *AKT1* and *BRCA1* mutations were shown to be independent prognostic factors, however this result needs to be further validated by the larger sample sizes included. Dysregulated PI3K and AKT signaling is considered one of the most common oncogenic changes in TNBC^23^. Recent randomized clinical trials have shown that AKT inhibitors in combination with first-line chemotherapy drugs prolong both progression-free survival (PFS) and overall survival (OS), and this benefit is more pronounced in patients with PIK3CA/AKT1/PTEN-altered TNBCs^24,25^. It is suggested that *AKT1* mutations are potential targets for the treatment of TNBC patients. In addition, *BRCA1* plays a role in several cellular pathways that maintain genomic stability^26^. Gaceb et al. reported that BRCA1 pathogenic variants were associated with poor prognosis, which is consistent with our findings^27^. Notably, PARP inhibitors are effective for treating tumors with *BRCA1/2* mutations^28^, suggesting that PARP inhibitors may be useful for treating TNBC patients.

The TNBC subtypes showed distinct genetic features and potentially actionable alterations. Patients with the LAR subtype showed high levels of *PIK3CA* mutations, with predominant enrichment in PI3K oncogenic pathways. This suggests that the LAR subtype may be sensitive to inhibitors of the PI3K pathway, such as PI3K/AKT/mTOR inhibitors. Randomized clinical trials of inhibitors targeting this pathway for the treatment of TNBC are currently underway. The mTOR inhibitors temsirolimus (DAT) and everolimus (DAE) were found to have notable objective responses in mesenchymal TNBC patients with PI3K pathway aberrations^29^. The results for alpelisib, a selective PI3Kα inhibitor, were also encouraging, showing significant efficacy against TNBC together with manageable toxicity when combined with nab-paclitaxel^30^. Patients with the LAR subtype of TNBC who received the PI3Kα-specific inhibitor taselisib plus enzalutamide showed improved clinical benefit in comparison with other TNBC subtypes^18^, indicating that the PI3K/AKT/mTOR pathway may be responsible for the biological changes in patients with the LAR subtype.

The BLIS subtype was observed to have the greatest number of CNVs, suggesting a high degree of genomic instability. Most BLIS samples (74%) were found to be 8q24.21amp and low-CIN. In addition, signature 19 was found only in the BLIS subtype, providing direction for subsequent studies on potential molecular features. In this study, no agents were found to potentially treat patients with the BLIS subtype, which may account for the poor prognosis of this subtype.

The IM subtype was enriched with *BRCA2* mutations and *MLL3* mutations. Mutations in the epigenetic regulator MLL3 were observed to be key drivers of the hybrid epithelial/mesenchymal phenotype and metastasis in breast cancer^31^. Loss of *MLL3* in vivo improved response to lapatinib in breast cancer^32^. It is suggested that *MLL3* could be a potential therapeutic target for patients in IM subtype. The subtype was also characterized by a predominance of mutations in the Notch pathway. Notch activation leads to proinflammatory cytokine production and the presence of tumor-associated macrophages in the tumor microenvironment^33^. Preclinical studies have observed reductions in Notch signaling after treatment with G9, a small-molecule USP9x inhibitor, together with remodeling of the tumor-associated immune environment and decreased tumor growth, with minimal toxicity^34^. It is suggested that patients in the IM subtype are potentially sensitive to Notch inhibitors. Notably, the present study revealed that patients with the IM subtype were all in the PD-L1 ≥ 1% subgroup and thus might be responsive to ICIs, which block immunosuppressive receptors, such as cytotoxic T lymphocyte antigen-4 (CTLA-4) and PD-1, to improve the cytotoxicity and proliferation of TILs^35^. The use of PD-1/L1 inhibitors together with chemotherapy has been found to be relatively successful in TNBC patients^36–38^, suggesting that ICIs may be an appropriate choice for patients with the IM subtype.

Tumors of the MES subtype were observed to be enriched in the RTK-RAS pathway and thus may have potential sensitivity to RTK-pathway inhibitors. Regorafenib, a multi-RTK inhibitor, has been shown to inhibit TNBC cell metastasis by targeting the SHP-1/p-STAT3/VEGF-A axis^39^. A small-molecule inhibitor cabozantinib (XL184) was also observed to significantly reduce multicellular invasive outgrowths in preclinical TNBC models^40^. This provides direction for future clinical trials targeting personalized therapy for MES patients.

There are several limitations to this study. First, the low proportion of MES subtypes may be related to the rarity of MES subtypes. This issue could be clarified using larger patient cohorts. Second, the study used only genomic data. Multi-omic data would allow a more comprehensive and in-depth investigation of TNBC heterogeneity and molecular features. Lastly, the study was retrospective and descriptive, and the results require confirmation in clinical settings.

## Conclusions

To sum up, the findings caution against the use of one-size-fits-all management of patients with TNBC. The TNBC subtypes were found to have unique molecular characteristics and, consequently, require specific therapeutic management. These findings assist the elucidation of the mechanisms underlying disease progression and thus contribute to its clinical management. Nevertheless, further prospective studies are required for further validation.

## Materials and methods

### Patients and samples

Patients with TNBC were retrospectively enrolled. The inclusion criteria were (1) aged at least 18 years, with confirmation of TNBC according to the guidelines of the American Society of Clinical Oncology-College of American Pathologists, (2) had not undergone neoadjuvant therapy, and (3) had provided written informed consent. Patients with multicentric or bilateral lesions were excluded. A total of 71 patients with TNBC who had undergone surgery at the Department of Breast Surgery at Shanxi Cancer Hospital between January 1, 2017, and December 31, 2019, were included. All patients received four to six cycles of anthracycline-based chemotherapy postoperatively. Resected specimens were fixed in formalin and paraffin-embedded (FFPE) and sections were evaluated separately by two experienced pathologists (RQL and JL). In the event of no consensus on the diagnosis, the case was reviewed by a third pathologist (HXM), and a majority vote was obtained. This study was approved by the institutional review board of Shanxi Cancer Hospital (Shanxi, China) (Approval Number: 2019075). We confirmed that all experiments were performed in accordance with relevant guidelines and regulations.

### Hematoxylin and Eosin (H&E) staining

After fixing the tumor tissue samples in 10% formalin, and cut into 5-μm slices, the sections were stained with hematoxylin and eosin (G1005, Servicebio, China) for H&E staining.

### IHC

An automated immunohistochemistry system was used to conduct IHC analyses of FFPE samples through a two-step approach in which a primary antibody was applied, followed by the application of a polymeric conjugate consisting of multiple secondary antibodies directly linked to a dextran backbone. All of the antibodies used for IHC analyses and data interpretation are summarized in Table S1. Staining was conducted as per the manufacturer’s protocols using a Roche automated IHC instrument (USA). All IHC protocols and antibodies used herein were included in a standard IHC panel used in the pathology laboratory.

### Identification of TNBC molecular subtypes and clinical characteristics

TNBC molecular subtyping was analyzed using histomorphological features, including apocrine differentiation, the presence of tumor-infiltrating lymphocytes (TILs), and metaplastic characteristics, as well as IHC markers including CD8, AR, and FOXC1. The details can be found in our previous publication^13^. Specifically, the 71 patients were classified into the four subtypes as follows: (1) LAR, AR+; (2) IM, high TIL levels, AR−, CD8+; (3) BLIS, low TIL levels, AR−, CD8−, FOXC1+; (4) MES, metaplastic features, AR−, CD8−, FOXC1−. Patients were also stratified into clinical groups based on their immune phenotype (immune-desert, immune-excluded, or inflamed), level of PD-L1 staining (< 1% or ≥ 1%), and growth pattern (infiltrative or expansive growth).

### Targeted NGS and alteration identification

Targeted NGS of 1021 genes was performed on 71 FFPE sections of TNBC specimens. DNA was extracted from the tissue using FirePureTM FFPE gDNA Extraction Kit and ultrasonicated (UCD-200, Diagenode, Seraing, Belgium) with fragment selection using Hieff NGS DNA selection beads. The DNA was quantified using a Qubit 2.0 Fluorometer with a Quanti-IT dsDNA HS Assay Kit (Thermo Fisher Scientific, MA, USA) and libraries were prepared using a custom apturing probe (IDT, IA, USA). Samples with library concentrations ≥ 20 ng/μL were sequenced using Paired-End 100 bp (PE100) with the Geneplus-2000 sequencing platform (Geneplus, Beijing, China).

The raw reads were then filtered with fastp (version 1) for removal of (a) reads with adaptors, (b) reads with N base proportions > 10% of the total lengths (unsure bases), and (c) single-end reads with low-quality base proportions > 50% of the total lengths (Phred scores < 5). Samples having ≥ 80% high-quality reads (Phred scores > 30) were retained for sequencing. After sequencing, the reads were compared with the hg19 reference human genome using the default parameter of BWA 0.6.2. Duplicates were identified by unique identifiers (UIDs) and positions of templates to avoid the introduction of errors by sequencing or PCR. GATK (V4.1.4.1) was used to identify single nucleotide variants (SNVs) and small insertions and deletions (InDels), together with performing quality control. Mutect 2.0 was used to analyze somatic SNVs and InDels in tumor tissues and copy number variations (CNVs) were identified by CNVKit. The self-developed algorithm NCsv (0.2.3) was used for the assessment of structural variation (SV)^41^. Synonymous variants, documented germline variants in matched gDNA and dbSNP, and variants with population frequencies > 1% in the Exome Sequencing Project were removed.

MSIsensor was used for the analysis of microsatellite instability (MSI)^42^, using an MSI score of > 19.5% as cut-off (MSI-H). The tumor mutation burden (TMB) was determined by assessing SNVs, small InDels, and variant allele frequencies (VAF) ≥ 1%. Higher TMB (TMB-H) was defined as ≥ 9 mut/MB.

### Genome characteristics and prognostic analysis

SNV data were used as input data. The “oncoplot” function in the “maftools” package in R was used for visualization of the mutation landscape and clinical features of the patients^43^. In addition, the “somaticInteractions” function was used for the identification of co-occurring or mutually exclusive genes using pairwise Fisher's exact tests. The patients were then allocated to wild-type or mutant-type gene groups based on the presence of nonsynonymous mutations and Kaplan–Meier (KM) analysis was used to compare disease-free survival (DFS). Multivariable Cox proportional hazard models were used to calculate the hazard ratios and 95% confidence intervals (CIs). Two-tailed p-values < 0.05 were considered statistically significant.

### Mutated pathway analysis

SNV data were used as input data and gene sets associated with pathways related to the DNA damage response (DDR) were acquired Wang et al.^44^. Pathways were considered mutated if they contained DDR-related genes with nonsynonymous mutations. The rates of mutations in these pathways were then evaluated and compared. The “OncogenicPathways” function in “maftools” was used for determining the numbers and proportions of mutations within ten known oncogenic pathways in TCGA. The enrichment of mutated cancer-related genes was analyzed using ClusterProfiler (version 3.12.0)^45^. The mutated genes and pathways were further analyzed using the KEGG and REACTOME databases^46–49^ with the calculation of p-values according to the hypergeometric distribution with correction of the false discovery rate (FDR) by the Benjamini and Hochberg method.

### Mutant-Allele Tumor Heterogeneity (MATH) scores and PyClone analysis

MATH scores are quantitative assessments of tumor heterogeneity and calculate the VAF distribution width^50^. The MATH scores for the tumor samples were determined using the “maftools” package in R. The clonal population structures of the tumors were analyzed using PyClone^51^ which assesses clonal structures through the grouping of SNVs with comparable frequencies together. Clusters with large mean cancer cell fractions (CCFs) were considered clonal.

### Mutational signature analysis

Mutational signatures from the Catalogue of Somatic Mutations in Cancer (COSMIC) were used. These utilize the proportions of the different types of substitutions, namely, C > A, C > G, C > T, T > A, T > C, and T > G, and their trinucleotide contexts, namely, the nucleotides preceding and following the mutated bases^52^. The combinations of COSMIC signatures were determined using the “deconstructSigs” package in R using an iterative approach^53^. Specifically, the appropriate structure of the input data was constructed using the “mut.to.sigs.input” method within the “deconstructSigs” package. The “whichSignatures” method was then used to identify the COSMIC signatures in the samples, as well as the contribution of individual signatures to the overall mutational spectrum.

Furthermore, R package “SomaticSignatures” uses non-negative matrix factorization (NMF) to identify de novo discovery of mutational signatures that are present within the different TNBC subtypes, and their contribution to each tumor’s mutational spectrum^54^. The 3-nucleotide mutation context of each SNV is extracted using the “mutationContext” method in the “SomaticSignatures” package. This function compares the locus of each SNV with the corresponding reference genome BSgenome.Hsapiens.UCSC.hg19 to identify the 3′ and 5′ of the SNV. Next, the frequency of each of the 96 alteration types was calculated using the “motifMatrix” method. “AssessNumberSignatures” was used to determine how many signatures we expected to recognize.

### CNV analysis

Analysis of CNVs was performed with GISTIC2.0 using default parameters (with a confidence level of 0.99). Visualization of the results was performed using “maftools” in R^55^.

### Mutational signature-based unsupervised clustering and CNV-based unsupervised clustering

Mutational signature clustering was determined by k-means clustering, using the “kmeans” R function. The “weight” of each signature, calculated by “deconstructSigs” was used as the input data for the samples. CNV-based clustering was determined by k-means and consensus clustering using the R package “ConsensusClusterPlus” to calculate the optimal number of subtypes. Input data for the samples were the “actual copy change given” of each “peak region” obtained from the file “all_lesions.conf_99.txt”. Enrichment of CNVs in the CNV-based subtypes was confirmed using Kruskal–Wallis tests.

### Analysis of putative clinically relevant alterations

Somatic SNVs/Indels and CNVs were analyzed by Precision Heuristics for Interpreting the Alteration Landscape (PHIAL) software (version 1.0.R) with default parameters and database.

### Statistical analysis

Student’s t-test, analysis of variance, and the Kruskal–Wallis test were utilized to compare continuous variables and ordered categorical variables whilst Pearson’s chi-square test and Fisher’s exact test were employed for comparison of unordered categorical variables. Survival curves were constructed using the Kaplan–Meier product limit method and compared with the log-rank test. A Cox proportional hazard regression model adjusting for available prognostic clinical covariates was performed to calculate HR and 95% CIs. The p values were adjusted to FDR using the Benjamini–Hochberg procedure in multiple comparisons. All analyses were performed using R packages version 3.4.2.

### Ethics approval and consent to participate

This study was approved by the institutional review board of Shanxi Cancer Hospital (Shanxi, China), and written informed consent were obtained (Approval Number: 2019075). We confirmed that all experiments were performed in accordance with relevant guidelines and regulations.

### Supplementary Information

Supplementary Figures.

Supplementary Tables.

## Data Availability

All data supporting the findings of this study are available within the manuscript and its supplementary information.
