# Supplementary material for: Genomic characterization reveals distinct mutational landscapes and therapeutic implications between different molecular subtypes of triple-negative breast cancer
Source: Sci Rep. 2024 May 29;14:12386. doi: 10.1038/s41598-024-62991-3 (PMC11137060; doi:10.1038/s41598-024-62991-3)
Supplement: Supplementary file 1 — Supplementary Figures. [file 41598_2024_62991_MOESM1_ESM.docx]

**Supplemental Figures For: Genomic Characterization Reveals Distinct Mutational Landscapes and Therapeutic Implications Between Different Molecular Subtypes of Triple-negative Breast Cancer**


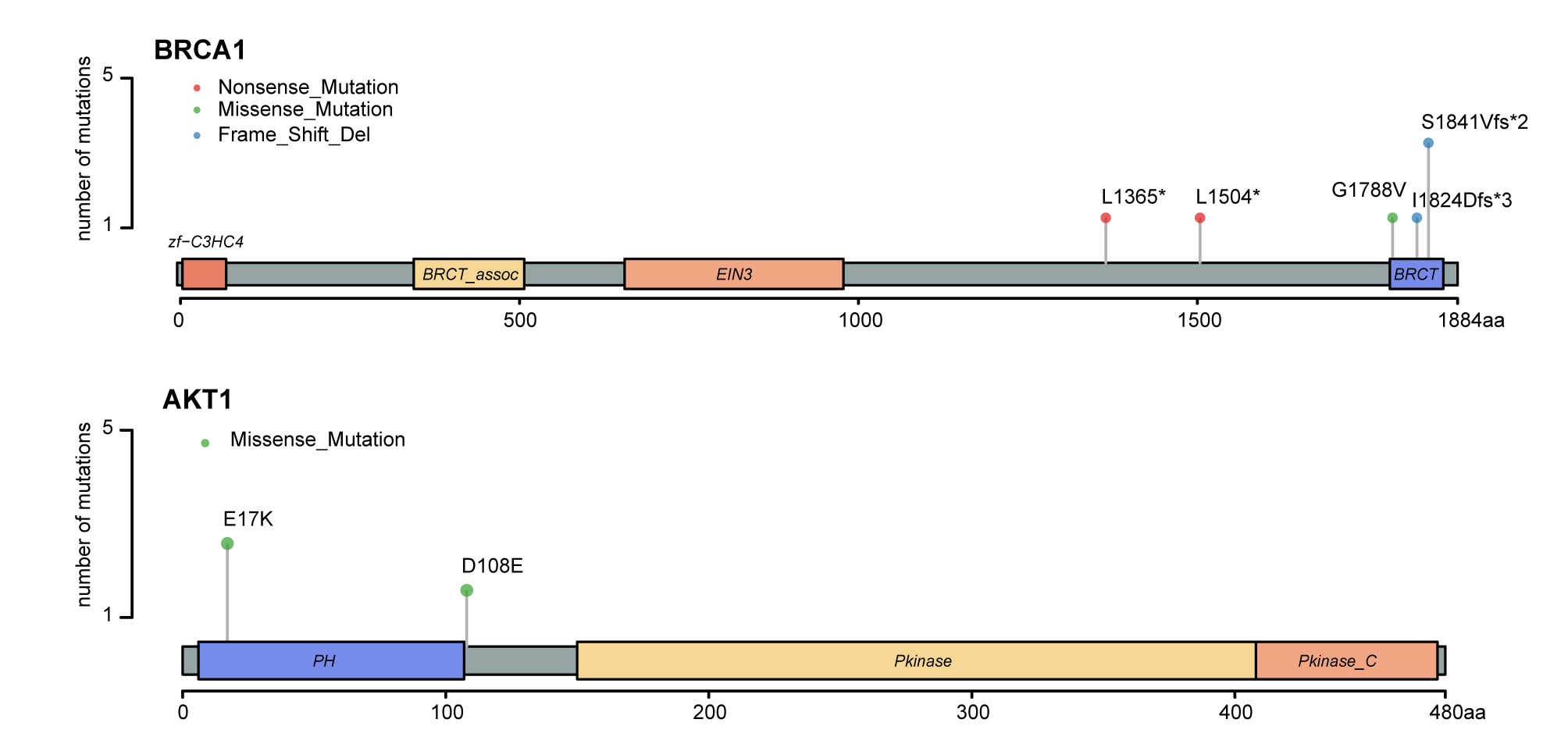


**Figure S1. Landscape of BRCA1 and AKT1 SNVs in TNBC.**

Different colors indicate mutation types and domains.


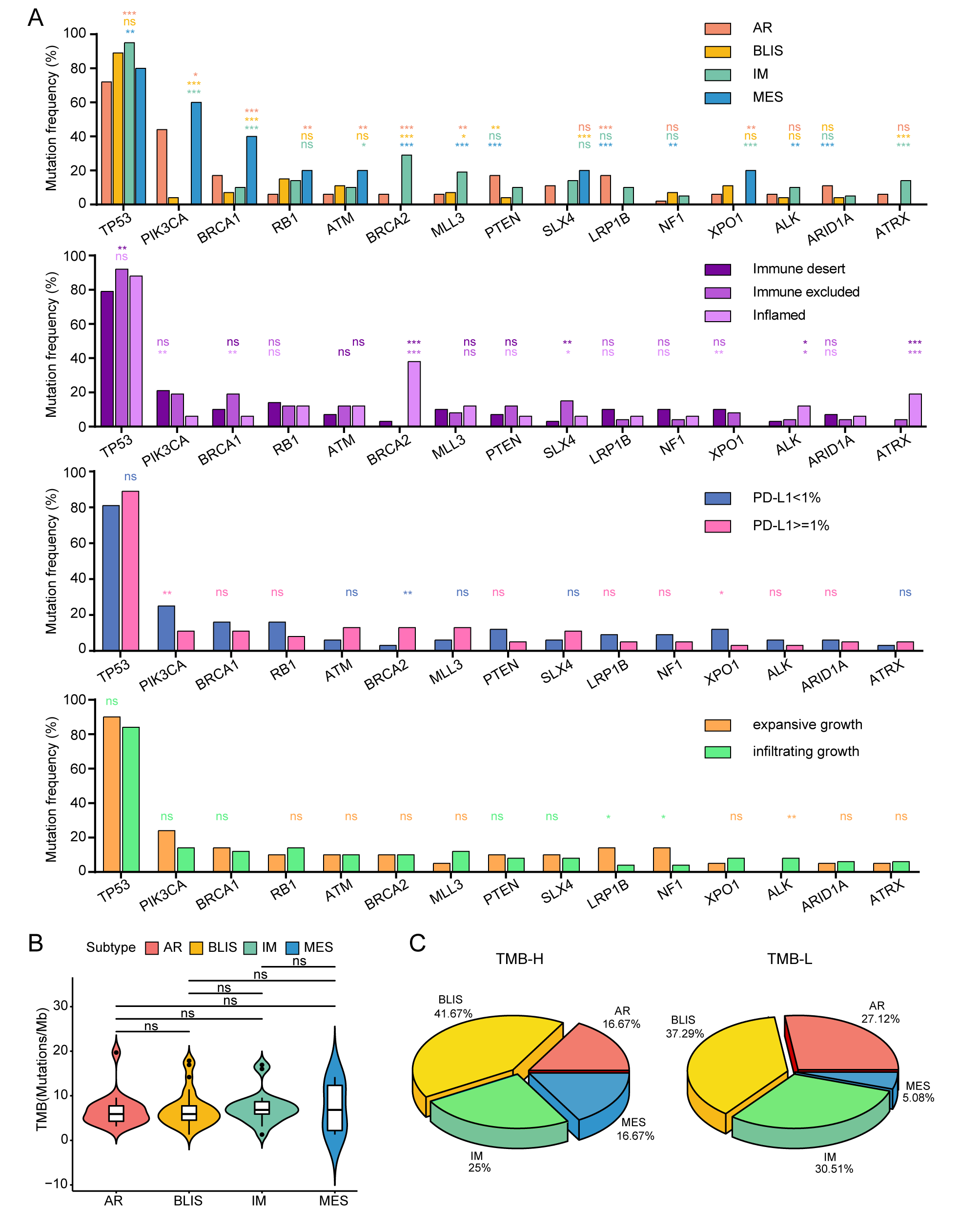


**Figure S2. Characteristic mutations in different groups.**

1. Frequency of mutation in top 15 mutated cancer-related genes. (B) TMB values in TNBC subtypes. (C) Proportions of TNBC subtypes in the high TMB (TMB-H) and low TMB (TMB-L) groups. Abbreviations: LAR, luminal androgen receptor; BLIS, basal-like immune-suppressed; IM, immunomodulatory; MES, mesenchymal-like.

**
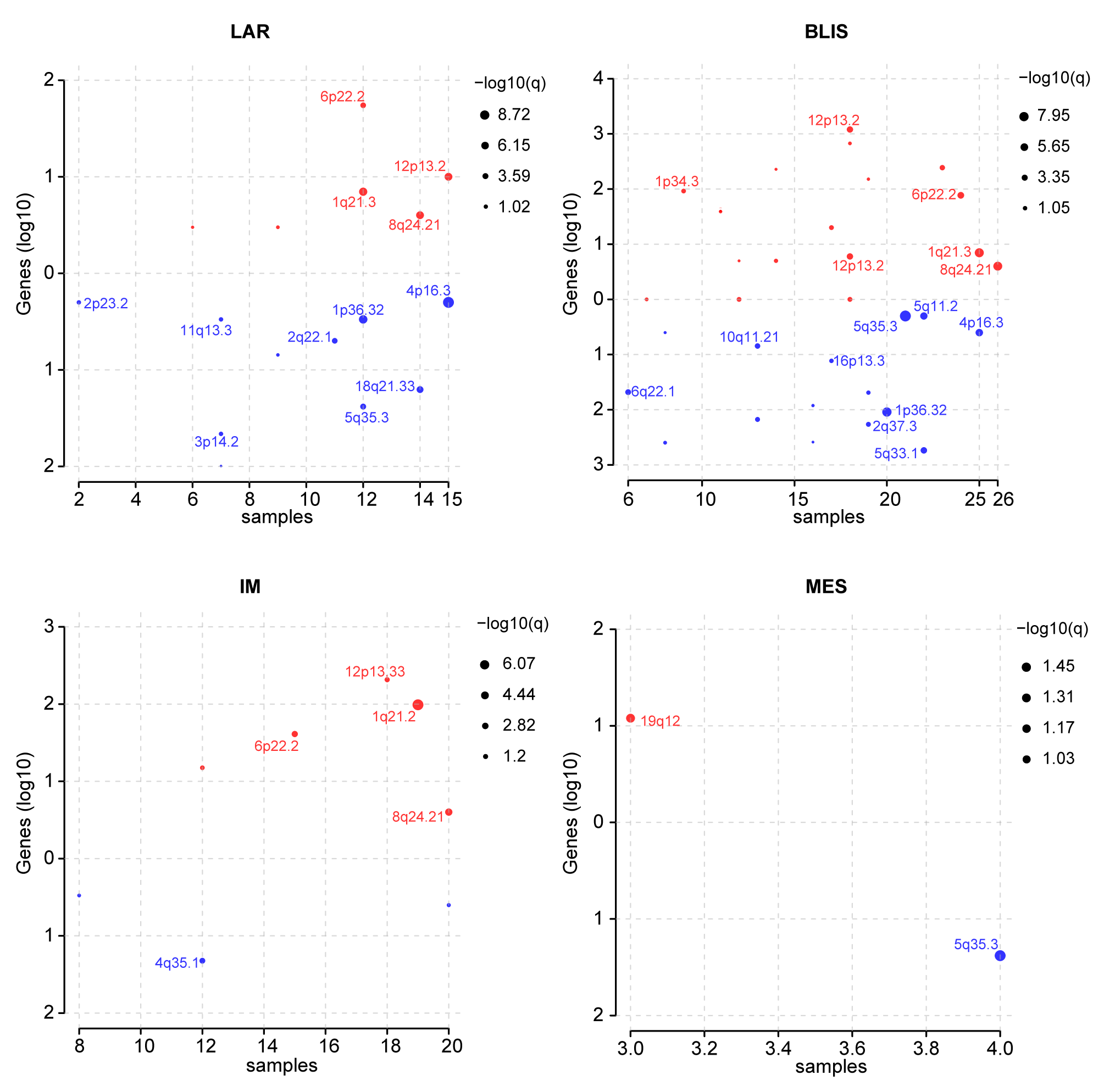
**

**Figure S3. Bubble plots showing gene numbers and sample sizes in significant CNV regions in TNBC molecular subtypes.**

Abbreviations: LAR, luminal androgen receptor; BLIS, basal-like immune-suppressed; IM, immunomodulatory; MES, mesenchymal-like.


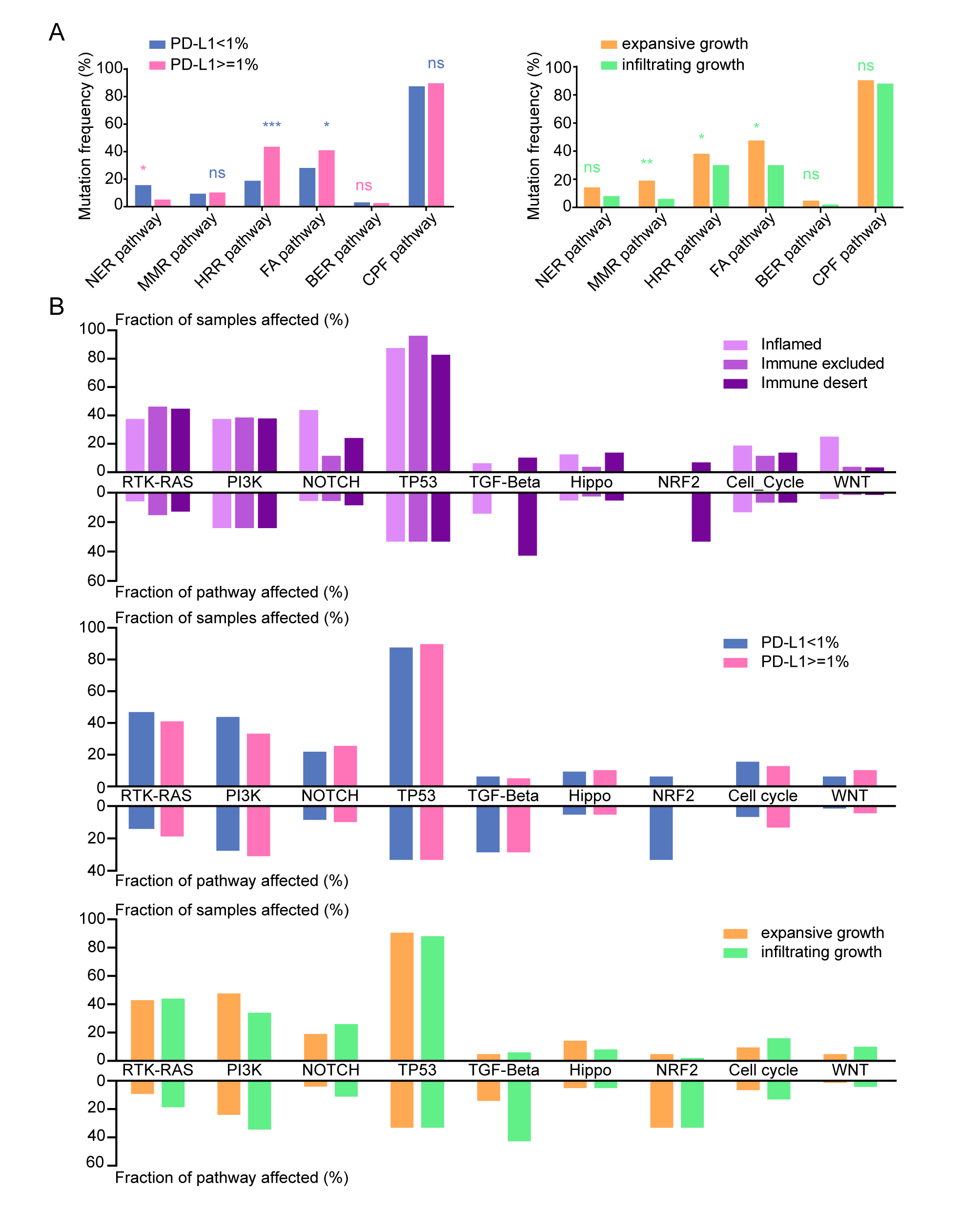


**Figure S4. Key signaling pathways affected by TNBC-associated somatic mutations.**

(A) Mutation rates in DDR-associated pathways in TNBC subgroups. (B) Proportions of affected oncogenic pathways in TNBC subgroups. Abbreviations: NER, nucleotide excision repair; MMR, mismatch repair; HRR, homologous recombination repair; FA, fanconi anemia; BER, base excision repair; CPF, checkpoint factor.


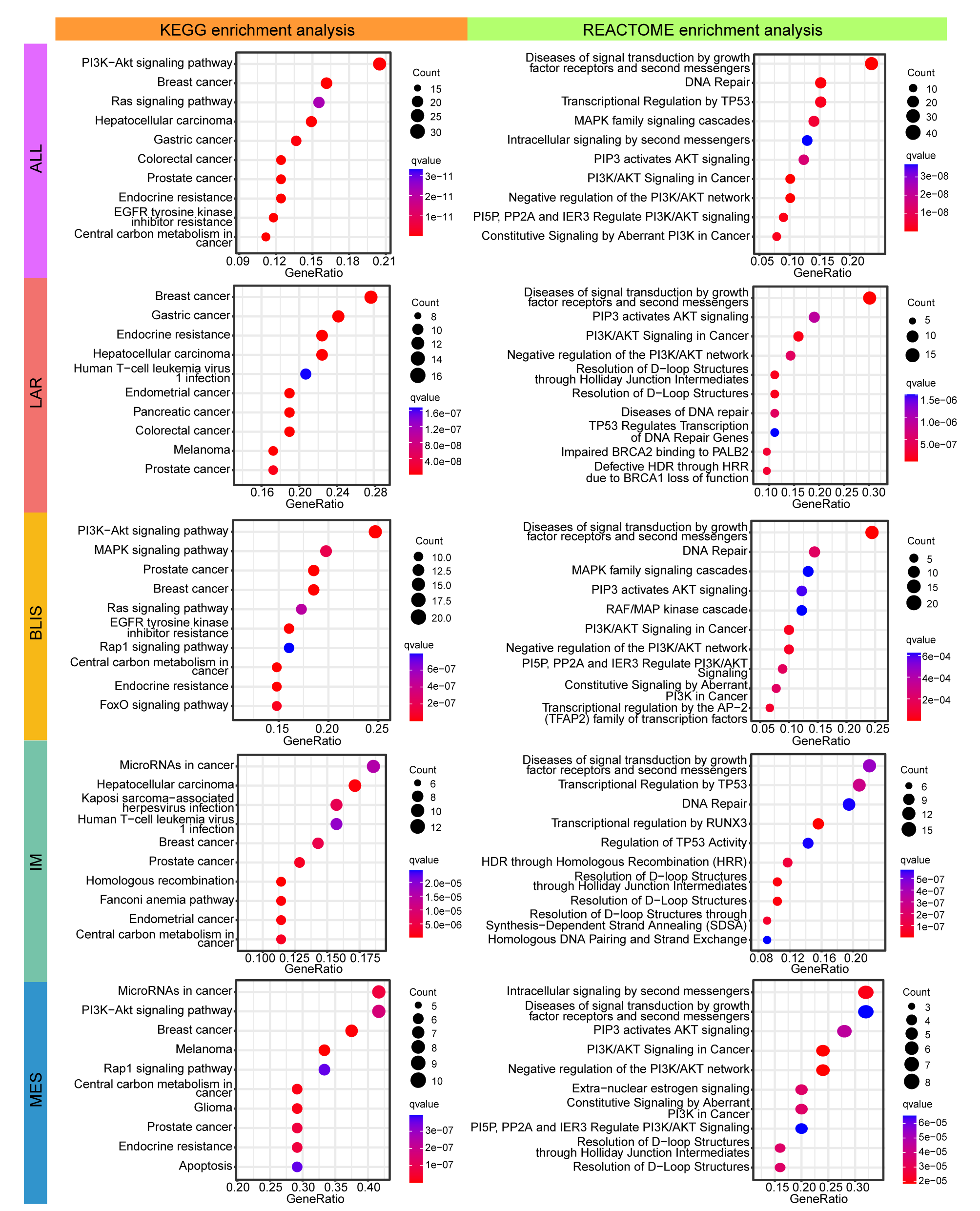


**Figure S5. KEGG and REACTOME enrichment analysis of mutated cancer-associated genes in TNBC subtypes.**

Abbreviations: LAR, luminal androgen receptor; BLIS, basal-like immune-suppressed; IM, immunomodulatory; MES, mesenchymal-like.


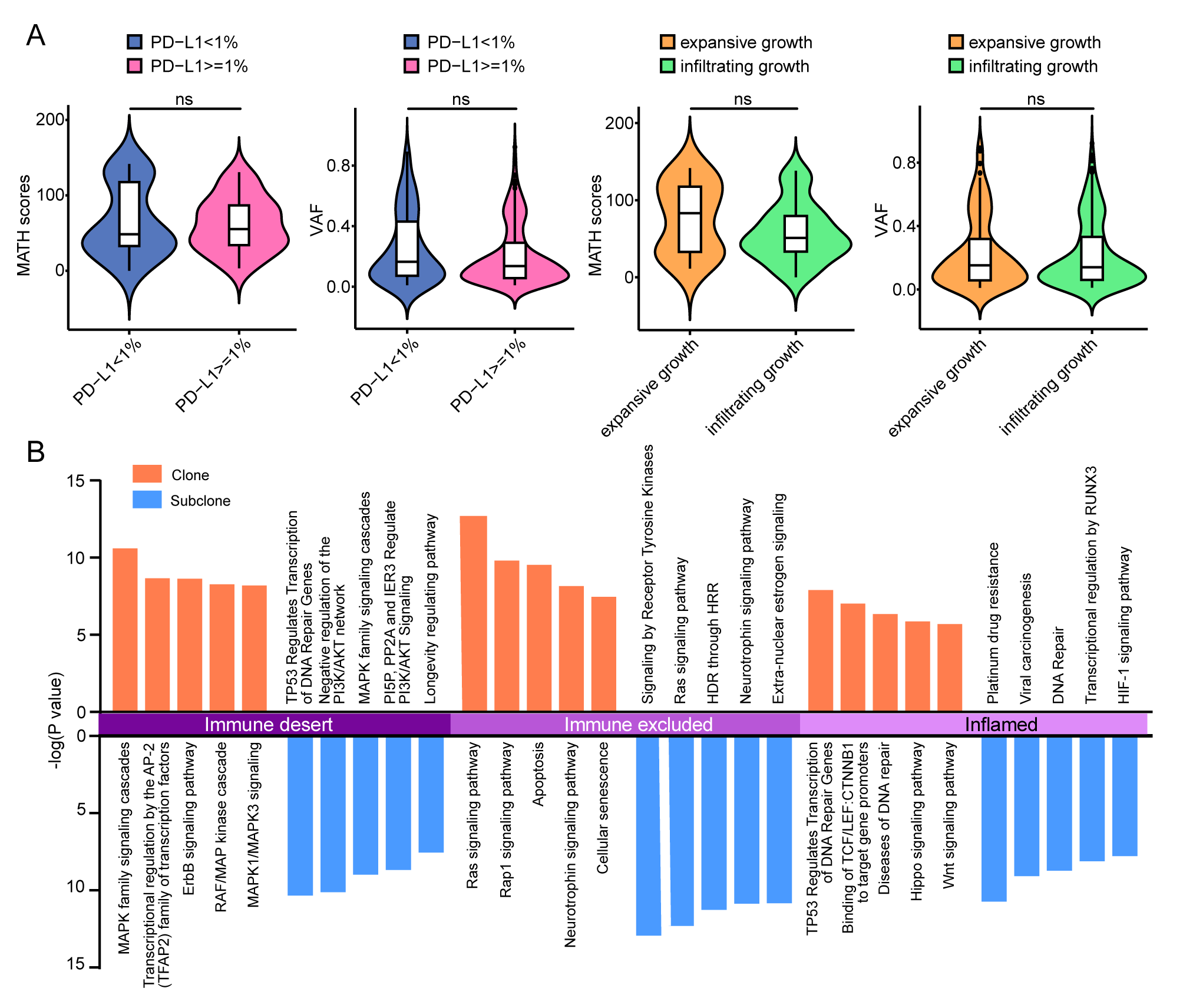


**Fig**u**re S6. Mutation heterogeneity and allelic mutation frequencies in TNBC.**

(A) Violin plot showing MATH scores and VAF in subgroups. (B) Enriched pathways associated with clonal and subclonal genes in different immune phenotypes. Abbreviations: MATH, mutant-allele tumor heterogeneity; VAF, variant allele frequency.


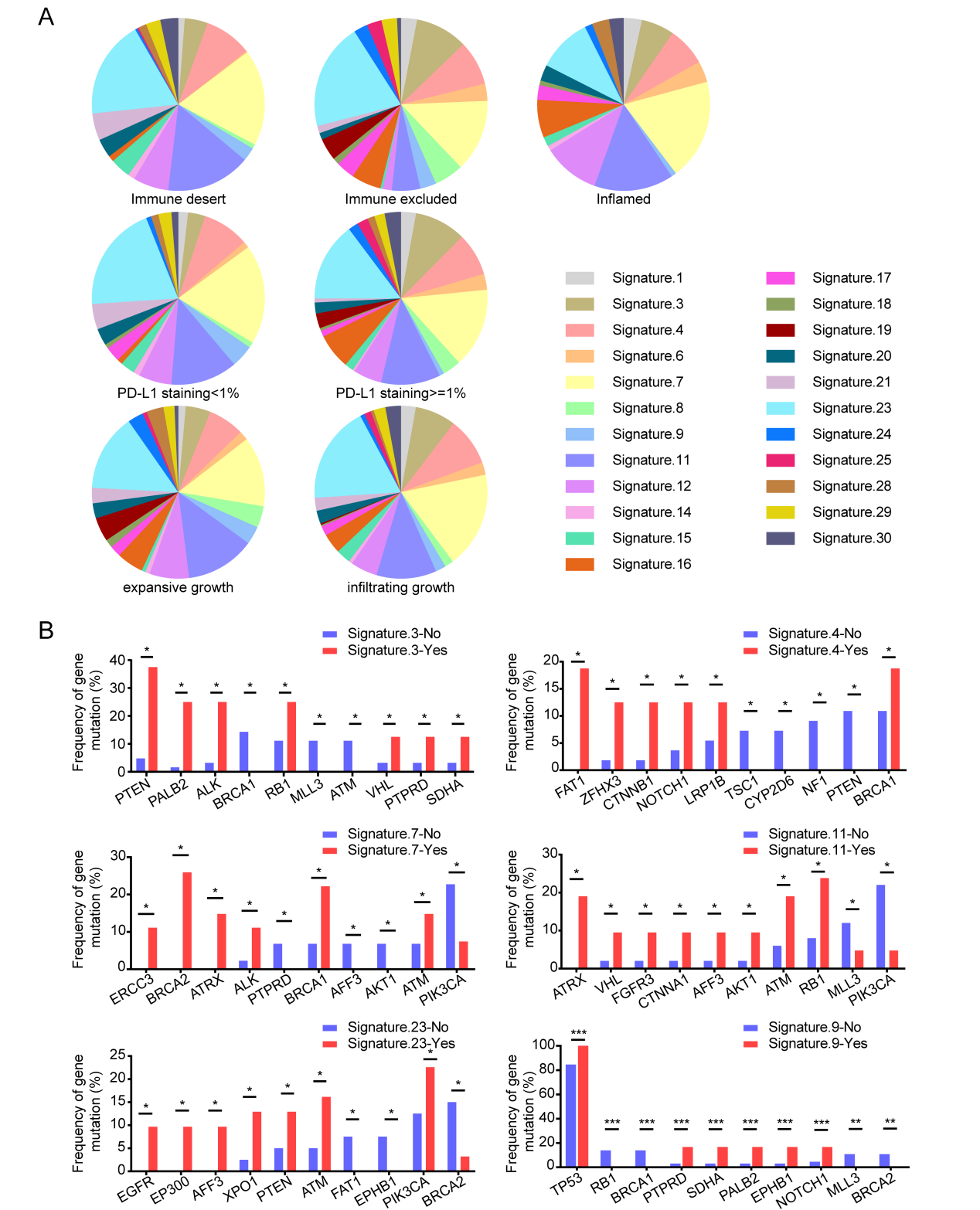


**Figure S7. The composition of mutation signatures.**

1. The composition of mutation signatures in different subgroups. (B) Frequencies of mutations in 10 top mutated cancer-associated genes in patients with or without signatures 3, 4, 7, 11, 23 and 9.


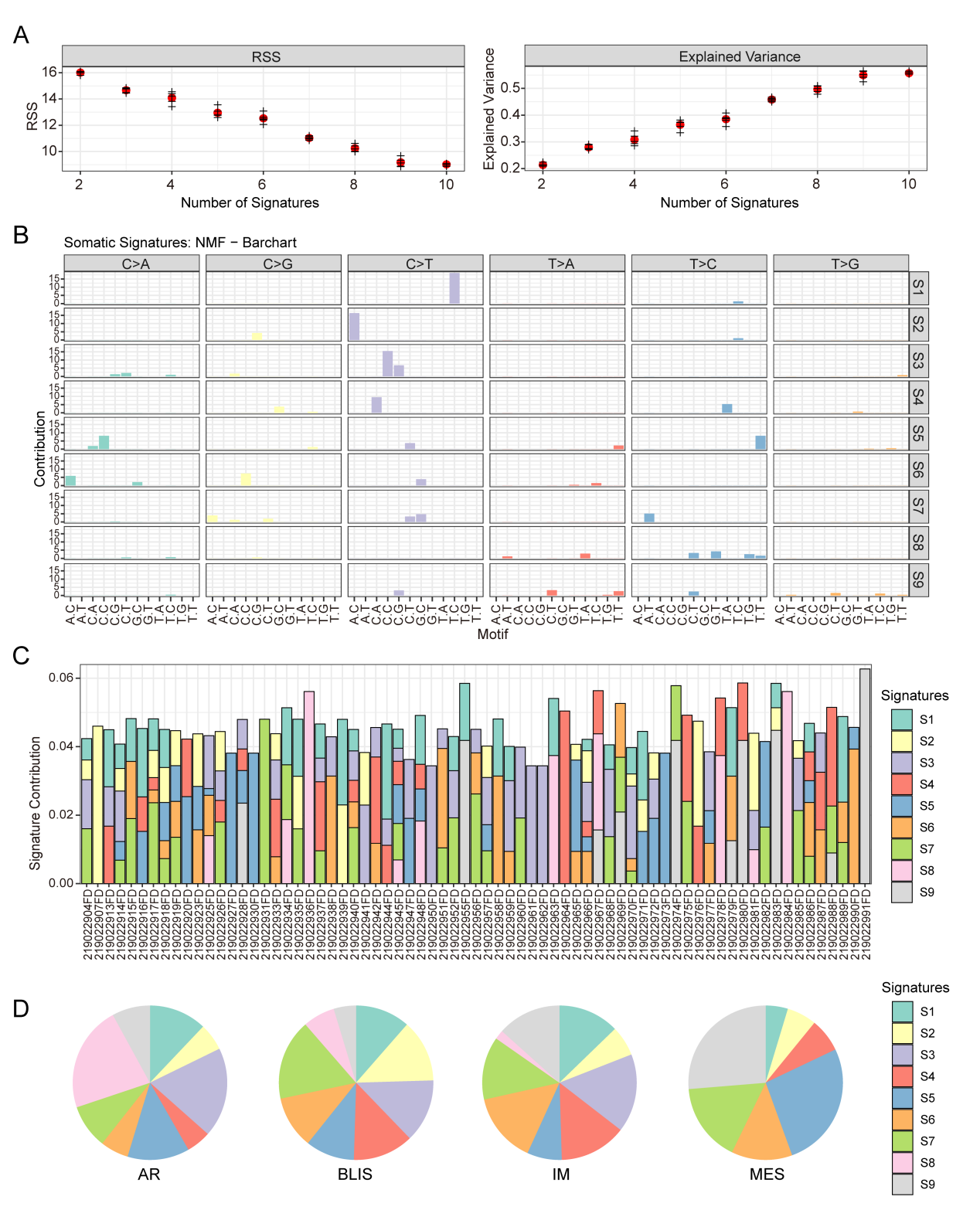


**Figure S8. Mutational signatures identified using the R package "SomaticSignatures".**

(A) The values of residual sum of squares (RSS) and explained variance when setting the number of signatures from 2 to 10. (B) Identifying nine mutational signatures in TNBC tumors using the R package "SomaticSignatures". (C) Contribution of nine mutational signatures in each TNBC patient. (D) Contribution of nine mutational signatures in different TNBC molecular subtypes.


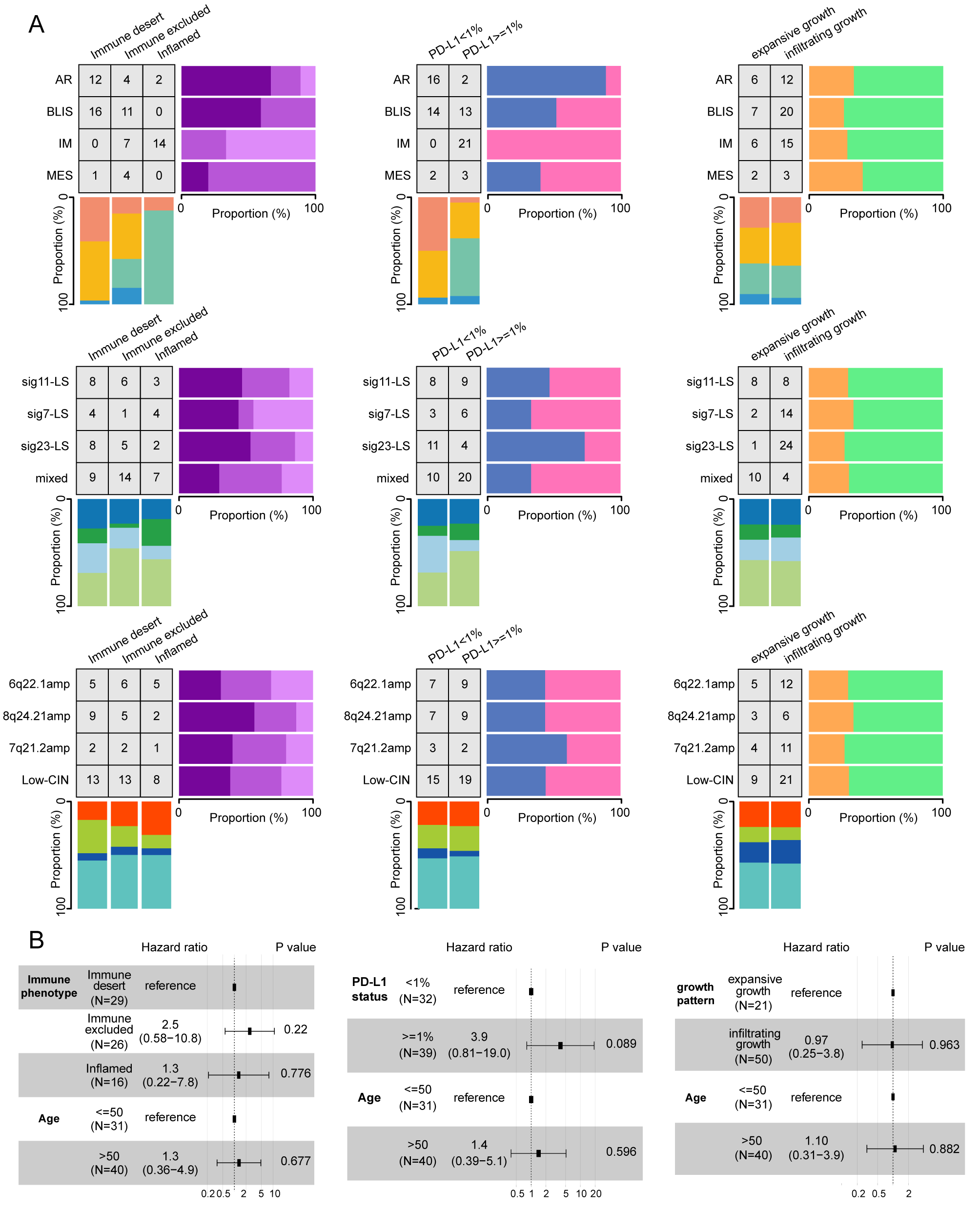


**Figure S9. Relationship between TNBC subtypes and clinical subgroups and impact on prognosis.**

(A) Relationships between TNBC subtyping and clinical grouping. (B) Association of immune phenotypes (left), PD-L1-staining (middle), and growth pattern (right) with DFS. Cox regression analysis with adjustment for age was used. Hazard ratios and 95% CIs are shown. Abbreviations: LAR, luminal androgen receptor; BLIS, basal-like immune-suppressed; IM, immunomodulatory; MES, mesenchymal-like.


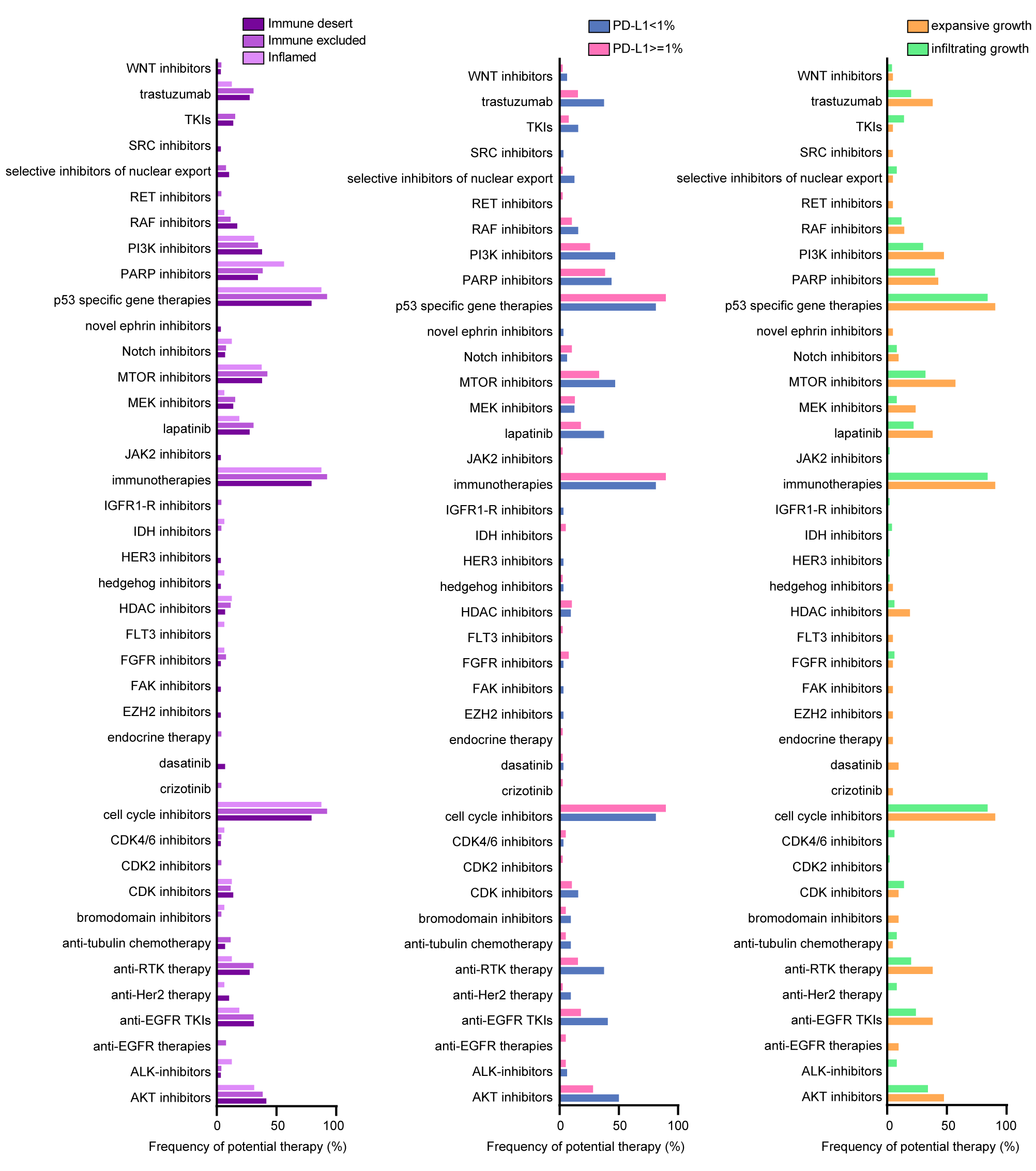


**Figure S10. Proportions of patients likely to be sensitive or insensitive to specific TNBC therapy in TNBC clinical subgroups.**
